# Treatment with a fixed dose combination antiretroviral therapy drug containing tenofovir, emtricitabine and efavirenz is associated with cardioprotection in high calorie diet-induced obese rats

**DOI:** 10.1371/journal.pone.0208537

**Published:** 2018-12-05

**Authors:** Frans Everson, Amanda Genis, Temitope Ogundipe, Patrick De Boever, Nandu Goswami, Amanda Lochner, Dee Blackhurst, Hans Strijdom

**Affiliations:** 1 Division of Medical Physiology, Faculty of Medicine and Health Sciences, Stellenbosch University, Cape Town, Republic of South Africa; 2 Environmental Risk and Health, Flemish Institute for Technological Research (VITO), Mol, Belgium; 3 Centre for Environmental Sciences, Hasselt University, Diepenbeek, Belgium; 4 Department of Physiology, Otto Loewi Research Center, Medical University of Graz, Graz, Austria; 5 Division of Chemical Pathology, Faculty of Health Sciences, University of Cape Town, Cape Town, South Africa; Azienda Ospedaliera Universitaria di Perugia, ITALY

## Abstract

HIV-infection, certain antiretroviral drug classes, especially protease inhibitors (PI), and obesity are associated with increased ischaemic heart disease (IHD) risk. However, the effect of PI-free fixed dose combination (FDC) antiretroviral therapy (ART) on hearts exposed to ischaemia-reperfusion injury (I/R) is unknown, particularly in obesity. This is becoming relevant as World Health Organisation guidelines recommend a FDC ART containing (non-) nucleoside reverse transcriptase inhibitors (tenofovir (TDF), emtricitabine (FTC) and efavirenz (EFV)) as first-line HIV treatment. Additionally, obesity rates are rising in HIV-infected populations, not only in ART-experienced individuals, but also at the time of ART initiation, which may further increase the risk of IHD. Therefore, we investigated the effects of PI-free FDC ART in myocardial I/R-exposed hearts from obese rats. Obesity was induced in male wistar rats *via* a 16-week high calorie diet. At week 10, treatment with a FDC ART drug containing TDF/FTC/EFV was initiated. Biometric and metabolic parameters, as well as myocardial functional recovery and infract size (IS), and myocardial signalling proteins following I/R were assessed after 16 weeks. Obese rats presented with increased body and intraperitoneal fat mass, elevated triglyceride and TBARS levels, whilst the hearts responded to I/R with impaired functional performance and increased IS. The FDC ART treatment did not alter biometric and metabolic parameters in obese rats. In a novel finding, ART protected obese hearts against I/R as shown by improved functional performance and smaller IS *vs*. untreated obese hearts. Cardioprotection was underscored by increased myocardial phosphorylated endothelial nitric oxide synthase (eNOS) and reduced AMP-kinase levels. In conclusion, these results demonstrate for the first time, that 6-weeks treatment of obese rats with a FDC ART drug specifically containing TDF/FTC/EFV conferred cardioprotection against I/R. The FDC ART-induced cardioprotection was seemingly unrelated to metabolic changes, but rather due to direct cardiac mechanisms including the up-regulation of myocardial eNOS.

## 1. Introduction

People living with HIV/AIDS are at higher risk of developing ischaemic heart disease and experiencing a future cardiac event compared to the general population [1–4]. The development of HIV-associated cardiac disease appears to be the consequence of viral and host factors, protease inhibitor (PI)-containing antiretroviral therapy (ART), and numerous other comorbidities [2,3,5]. In addition, obesity, a recognised modifiable ischaemic heart disease risk factor, is increasingly observed in HIV-infected populations, not only as a consequence of ART-induced reversal of HIV/AIDS associated weight loss [6–9], but interestingly also in HIV-infected individuals at the time of diagnosis before the initiation of ART [10,11]. These findings suggest that obesity rates in HIV-infected populations are either approaching or already mirroring those of the general population [7,8,12], which may pose an additional cardiac risk in such individuals. Although some antiretroviral drugs, most notably PIs, are associated with the development of cardiac and metabolic abnormalities [13–15], there is a paucity of data on the cardiac effects of PI-free, fixed dose combination (FDC) ART drugs specifically containing nucleoside transcriptase inhibitors (NRTI’s) and non-nucleoside transcriptase inhibitors (NNRTI’s), especially in the context of ischaemia-reperfusion (I/R) injury. Furthermore, the role of these specific ART combination drugs in I/R exposed hearts from obese subjects is not well described, particularly in subjects presenting with obesity at the time of ART initiation. These knowledge gaps are becoming clinically relevant, both in terms of the rising obesity rates posing potential additional cardiovascular risk, and recent World Health Organisation (WHO) guidelines that strongly recommend the use of once daily first-line FDC drugs consisting of two NRTI’s: Tenofovir (TDF) plus Lamivudine or Emtricitabine (FTC), and one NNRTI: Efavirenz (EFV) [16].

To address the above knowledge gaps, the overarching aim of the present study was to investigate the role of treatment with a specific FDC ART drug containing TDF, FTC and EFV in I/R exposed hearts from obese male wistar rats. Furthermore, biometric and metabolic parameters were assessed, and the expression of selected cardiac proteins measured to determine whether the drug effects in the hearts were due to metabolic changes or direct cardiac mechanisms. We chose a model of high calorie diet- (HCD) induced obesity, which has previously been shown in our laboratory to result in poorer outcomes in isolated perfused rat hearts exposed to I/R injury [17–19]. The total feeding programme was of 16 weeks’ duration, and ART treatment commenced in week 10 and continued for the final 6 weeks. At week 10, the weights of the rats receiving the HCD were significantly higher compared to the control animals, ensuring that the HCD animals had developed obesity at the time of ART initiation. Furthermore, the experimental design ensured that the effects of both obesity and FDC ART treatment were appropriately controlled for. This was achieved by the age- and time-matched inclusion of the following groups: untreated HCD-induced obese rats (receiving HCD and vehicle treatment: controlling for FDC ART), treated lean control rats (receiving standard chow and FDC ART treatment: controlling for obesity), and untreated lean control rats (receiving standard chow and vehicle treatment: controlling for obesity and FDC ART).

The main endpoints of the study included standard biometric and metabolic measurements in the animals, cardiac function and infarct development following I/R injury, and the measurement of important cardiac proteins associated with protection or damage. Biometric measurements (total body mass (TBM), intraperitoneal (IP) fat mass, heart and liver mass) were performed to characterize the obesity model and to assess the effects of FDC ART treatment on biometric parameters such as body and organ weights. To assess the effects of obesity and FDC ART treatment on metabolism and oxidative stress, the following parameters were measured: fasting blood lipids and glucose levels, as well as serum markers of lipid peroxidation (thiobarbituric acid reactive substance (TBARS) and conjugated dienes (CD)). These measurements additionally assisted in determining whether any of the observed ART-induced cardiac effects were related to metabolic mechanisms. The susceptibility of the hearts to an I/R insult was assessed with a well-characterized isolated rat heart perfusion model [20–22]. The effects of obesity and FDC ART in the hearts were determined by measurements of mechanical function and infarct size (IS) development. Finally, in order to determine whether obesity and FDC ART exerted their effects on the hearts via direct cardiac mechanisms, selected proteins, mainly derived from the ventricles, were measured. These included known cardioprotective signalling proteins, endothelial nitric oxide synthase (eNOS) and Akt [23–25], the prominent myocardial regulator of energy production, 5' adenosine monophosphate-activated protein kinase (AMPK) [26], nitrotyrosine, a marker of nitrosative stress [27], and p22 phox, a subunit of nicotinamide adenine dinucleotide phosphate (NADPH)-oxidase, which is a prominent source of myocardial reactive oxygen species production [28].

## 2. Materials and methods

### 2.1. Animal care

The study was approved by the Research Ethics Committee: Animal Care and Use, University of Stellenbosch (Protocol number: SU-ACUM13-00025), and complied with the “Guide for the Care and Use of Laboratory Animals” (US National Institute of Health: Publication 85–23, revised 1985) and the South African (SA) National Standards for the Care and Use of Animals for Scientific Purposes (South African Bureau of Standards, SANS 10386, 2008). Animals were housed in the Central Research Facility (Faculty of Medicine and Health Sciences, University of Stellenbosch) at 22°C, 40% humidity, subjected to a 12-hour artificial day/night cycle. All surgical procedures on animals were performed under full anesthesia and all efforts were made to minimize suffering.

### 2.2. Animals and groups

The overarching aims of the study were to determine whether treatment with a specific FDC ART (containing TDF, FTC and EFV) affects the function and infarct development in hearts from HCD-induced obese rats following I/R injury, and whether these responses are associated with changes in biometric and metabolic parameters, as well as cardiac-specific expression of selected proteins. To investigate these aims, 118 male wistar rats, approximately 8 weeks old, were randomly assigned to four groups: (i) vehicle treated lean control group (Control; *n* = 28), vehicle treated HCD-induced obese group (HCD; *n* = 28), ART treated lean control group (Control+ART; *n* = 28) and ART treated HCD-induced obese group (HCD+ART; *n* = 34).

### 2.3. Diet and feeding programme

Animals in all four groups had *ad libitum* access to standard rat chow and tap water. The obese groups additionally received a HCD rich in fat, carbohydrates and sucrose as previously developed and used in our laboratory [20,29]. The nutrient and energy composition of the standard rat chow *vs*. HCD were as follow (/100 g): total fat 4.8 g *vs*. 11.5 g; saturated fat 0.9 g *vs*. 7.6 g; cholesterol 2.2 mg *vs*. 13 mg; protein 17.1% *vs*. 8.3%; carbohydrates 34.6% *vs*. 42%; sucrose 5.3% *vs*. 20.4%; total dietary fiber 26.9 g *vs*. 9.3 g; and energy 1272 kJ *vs*. 1354 kJ [20,29]. The feeding programme was of 16 weeks’ duration. During the feeding programme, body weights were recorded individually on a weekly basis (every seven days during the morning) from week 1 to week 16 of the feeding programme. For the purposes of this study, obesity was defined as a significant increase in the mean TBM of animals receiving the HCD compared to the lean control animals receiving standard rat chow.

### 2.4. Drug treatment

A commercially available FDC ART drug, Odimune (Cipla, SA), containing TDF (300 mg), FTC (200 mg) and EFV (600 mg), was used for the study. To assess the effects of the FDC ART drug in animals that had developed obesity at the time of ART initiation, drug administration commenced in week 10 of the feeding programme at which time-point the rats receiving the HCD were significantly heavier than their control counterparts. Tablets were ground to a fine powder, and the required amounts weighed out according to recommended therapeutic doses for humans. Due to the increased metabolic rates exhibited by rats, we followed the recommendations of the US Food and Drug Administration according to which the equivalent drug dose should be ~6 times higher than the human dose [30]. Based on these calculations, rats in the treatment groups received TDF (25.8 mg/kg/day), FTC (17.4 mg/kg/day) and EFV (51.6 mg/kg/day). The powdered drug was suspended in 1 ml water and administered to each rat daily *via* oral gavage during the last six weeks of the 16-week feeding programme. Vehicle control animals received 1 ml of drug-free water daily *via* oral gavage in a time-matched fashion.

### 2.5. Experimental procedures

Following the 16-week experimental programme, animals were anaesthetized by IP sodium pentobarbitone (160 mg/kg) injection. Upon disappearance of the foot-pinch reflex, the rats were weighed and the TBM values recorded, after which the animals were sacrificed *via* a clamshell thoracotomy and exsanguination.

#### 2.5.1. Biometric measurements

In addition to the TBM measurements, the IP fat tissue (including epididymal, retroperitoneal and perirenal fat), liver and heart were removed from each animal and weighed post-euthanasia in a weighing boat using an electronic scale (Ragwag, Torunska, Poland).

#### 2.5.2. Metabolic and oxidative stress analyses

A number of rats (*n* = 5–6 / group) were randomly sampled for overnight fasting and subsequent biochemical analyses. Fasting blood glucose levels were determined by analysing a blood droplet obtained from a tail prick (during deep anaesthesia) with a commercially available glucometer (Gluco Plus, CIPLA DIBCARE, Bellville, SA). Furthermore, blood that pooled in the thoracic cavity following exsanguination was collected and serum extracted for subsequent measurement of the total cholesterol (TC), high-density lipoprotein cholesterol (HDL), triglyceride (TG), CD, and TBARS levels (Courtesy Dr. Dee Blackhurst, Division of Chemical Pathology, University of Cape Town, SA). TC and TG levels were determined spectrophotometrically (SPECTRA-max Plus 384 and SoftMax Pro 4.8 microplate data acquisition and analysis software; Labotec Industrial Technologies, SA) with enzymatic colorimetric kits (LabAssay Cholesterol; LabAssay Triglyceride; Wako Chemicals, Germany). HDL was also measured *via* a colorimetric assay, but a dual separation method was applied as previously described [31]. Finally, CD and TBARS concentrations were determined by spectrophotometry (SPECTRA-max Plus 384 as above) and calculated using the appropriate molar extinction coefficients. CD concentrations were calculated by means of absorbance (234 nm after appropriate dilution in cyclohexane (Spectrosol) as previously reported [32,33]. TBARS levels were determined by a method described by Jentzsch *et al*. [34], and concentrations determined *via* absorbance at 532 nm.

#### 2.5.3. Isolated heart perfusions

**Fig 1:**
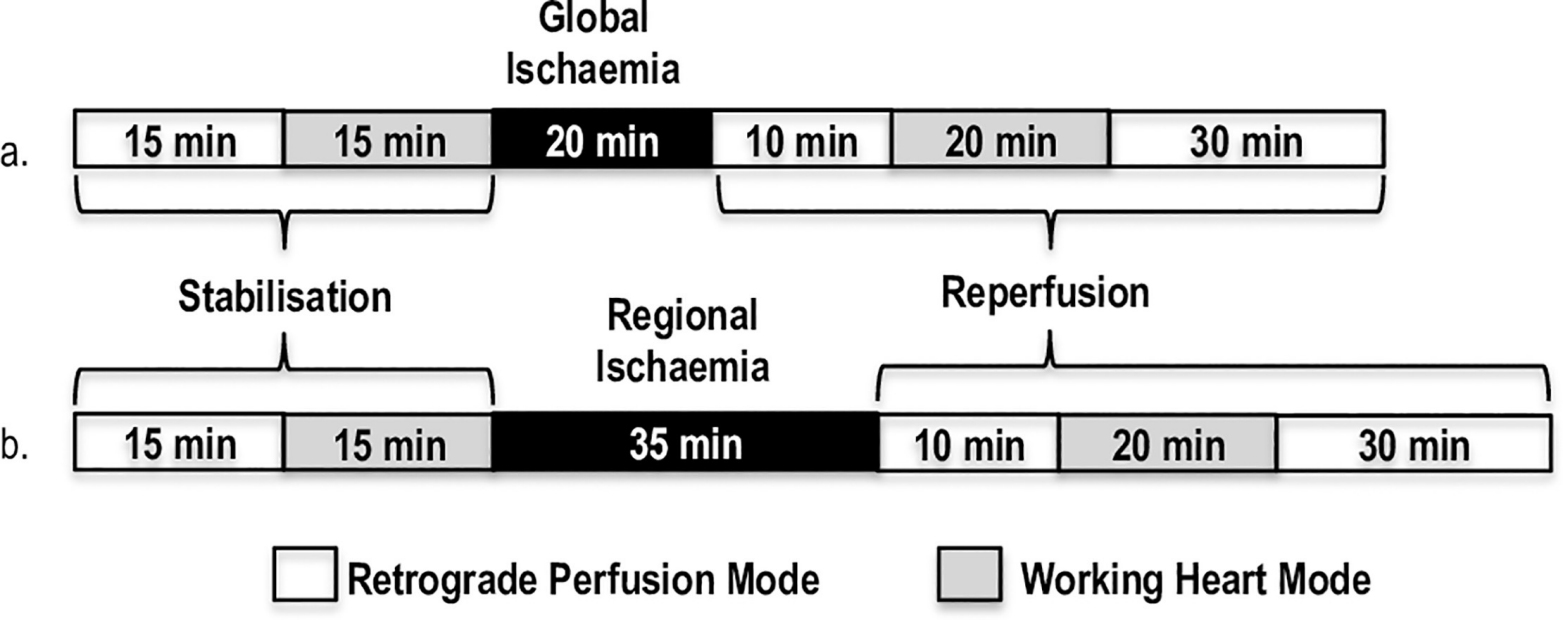
Isolated heart perfusion protocol: (a) Global, and (b) Regional I/R. Global ischaemia was induced by total cessation of perfusion to the myocardium through closure of both aortic and atrial cannulae for 20 min. Regional ischaemia was induced by ligation of the proximal left anterior descending coronary artery (LAD) with a silk suture for 35 min resulting in ~67% reduction in coronary flow.

After excision and removal of excess fat and connective tissue, hearts were immediately placed in ice-cold Krebs-Henseleit bicarbonate buffer solution (composition in mM: NaCl 119; NaHCO_3_ 24.9; KCl 4.7, KH_2_PO_4_ 1.2; MgSO_4_.7H_2_O 0.59; Na_2_SO_4_ 0.59; CaCl_2_ 1.25; glucose 10). Following this, the hearts were mounted on an *ex vivo* isolated heart perfusion system. The hearts were perfused normothermically (36.8 **°**C) with non-recirculating Krebs-Henseleit bicarbonate buffer, and continuously gassed (95% O_2_/5% CO_2_) through a sintered glass oxygenator (pH 7.4). Temperature was carefully controlled throughout the experiment by inserting a temperature probe into the left atrium. The aortic pressure development was obtained through a side branch of the aortic cannula, which was connected to a Viggo-spectramed pressure transducer. Coronary flow and aortic output were measured manually. Hearts were subjected to either global or regional I/R, and the protocols are shown in **Fig 1.** The global I/R protocol was used to measure myocardial mechanical function when the whole heart was exposed to zero perfusion, as well as to provide sufficient tissue to prepare ventricular homogenates for western blot analyses, while the regional I/R protocol allowed for the simulation of an acute myocardial infarction of the left ventricle and IS determination, as well as measurement of myocardial mechanical function in the regional I/R setting.

Pre-ischaemic and post-ischaemic myocardial functional performance was determined by measuring peak systolic pressure (PSP), coronary flow (CF), aortic output (AO), cardiac output (CO = CF+AO), and total work (Wt) at the end of the 30 min stabilisation period and at the end of the 20 minutes working heart phase of reperfusion respectively in hearts subjected to global and regional ischaemia. The Wt performance of these hearts was calculated according to the formula of Kannengieser and coworkers [35]. Only hearts with an AO of more than 34 ml/min at the end of the stabilisation period were used for Western blotting and IS determinations. Following regional I/R, the silk suture around the LAD was permanently tied and 0.25% Evans blue solution infused into the heart to outline the viable tissues. The IS was determined by means of tetrazolium staining as described previously [36]. Briefly, the ventricles were frozen, cut into 2 mm thick transverse slices and subsequently stained with 1% triphenyl tetrazolium chloride in phosphate buffer, pH 7.4, for 10 min. The left ventricular area at risk and infarcted area were measured by means of computerized planimetry (UTHCSA Image Tool programme, University of Texas Health Science Center at San Antonio, TX, USA). IS was expressed as a percentage of the area at risk.

#### 2.5.4. Western blot analysis

The expression and activation by phosphorylation of several proteins involved in important myocardial signalling pathways were measured *via* Western blotting protocol routinely used in our laboratory [22,36,37]. To assess proteins under baseline conditions, the ventricles of the hearts from the fasted animals (allocated for the biochemical analyses) were freeze-clamped post-euthanasia and stored at -80°C. These hearts were not destined for perfusion experiments, hence providing an opportunity to explore baseline protein status. In a second set of western blot analyses, signalling proteins were assessed in hearts that had been exposed to 20 min global ischaemia and freeze-clamped after 60 min reperfusion.

**Antibodies:** The following antibodies were purchased from Cell Signaling Technologies Inc. (Beverly, Massachusetts, USA): anti-eNOS (catalogue number: ♯9572), anti-phospho-eNOS (Ser1177; catalogue number: ♯9571), anti-Akt (PKB/Akt; catalogue number: ♯9272), anti-phospho-Akt (Ser473; catalogue number: ♯4060), anti-AMPK (catalogue number: ♯2532), anti-phospho-AMPK (Thr172; catalogue number: ♯2535), and anti- β-tubulin (catalogue number: ♯2146). The following antibodies were purchased from Santa Cruz Biotechnologies Inc. (Santa Cruz, California, USA): anti-p22-phox (FL-195; catalogue number: sc-20781) and anti-nitrotyrosine (PNK; catalogue number: sc-55256). (For more information on the antibodies see supporting figures.)

**Lysate preparation and analyses:** For the preparation of lysates, frozen cardiac tissue of mainly ventricular origin (approximately 30 mg/heart), previously stored in liquid nitrogen, was mechanically pulverized and fully homogenized for 2 x 5 seconds (Polytron PT10 homogenizer, Next Advance, Inc., New York, USA) in 800 μl ice-cold lysis buffer (20 mM Tris/1mM ethylene glycol tetraacetic acid, pH 7.4; 1 mM ethylenediaminetetraacetic acid; 150 mM NaCl; 1 mM β-glycerophosphate; 1 mM sodium orthovanadate; 2.5 mM sodium pyrohosphate; 50 μg/ml phenylmethylsulfonyl fluoride; 0.1% sodium dodecyl sulphate; 10 μg/ml aprotinin; 10 μg/ml leupeptinin and 1% triton-X100). The protein concentration was determined by the Bradford method [38]. Lysates were boiled and stored at -80° C. For Western blotting, equal amounts of proteins were separated on a sodium dodecyl sulphate poly-acrylamide gel and electro transferred to Immobilon^TM^-P membranes (Merck Pty Ltd., Darmstadt, Germany). The membranes were blocked with Tris-buffered saline (TBS) (pH: 7.6); 0.1% Tween-20 and 5% non-fat powdered milk) and incubated overnight in the primary antibodies. Ponceau-reversible staining or β-tubulin (after stripping the membranes with 2% NaOH for 5 minutes and re-probing with β-tubulin was used to validate protein transfer and equal loading as measured by densitometry software and subsequent statistical analysis confirming no significant differences between groups). For Western blot analyses on baseline hearts the light emitting from the luminescence was captured on medical x-ray film and the images analysed by densitometry software (UN-SCAN-IT: Silk Scientific Inc., Orem, UT, USA). The Western blot analyses of post-ischaemic hearts were performed by utilizing the Chemidoc MP Imager System with Image Lab- 5 software (BIO-RAD Inc., USA). The proteins were detected by covering the membranes with enhanced chemiluminescence detection reagent (ECL). Values were obtained and normalized to the mean of the untreated control group. All chemicals used in the Western blot analyses were obtained from Merck Chemical Co (Cape Town, SA). Horseradish peroxidase (secondary antibody), ECL and hyperfilm were acquired from Amersham, Life Sciences (Buckinghamshire, UK).

### 2.6. Statistical analysis

Weekly TBM during the feeding programme was expressed in gram and statistically analysed by means of a two-way analysis of variance (ANOVA) followed by a Bonferroni multiple comparison. Final TBM and biometric parameters, as well as metabolic and oxidative stress parameters were analysed *via* one-way-ANOVA (with Bonferroni multiple comparison). To elucidate the exclusive effect of the diet or ART treatment, independent of the other variables, a student’s t-test was used from time to time. IS was expressed as percentage of the area at risk and analysed by means of a one-way-ANOVA (with Bonferroni multiple comparison). Western blot data are expressed as–fold change or % change compared to control for increases and decreases respectively and statistically analyzed by means of a 1-way ANOVA, followed by Tukey’s multiple comparison. All data were analyzed with GraphPad Prism Version 7 (GraphPad Software, USA). Values are expressed as mean ± standard error of the mean (SEM). Statistical significance was set at a *p*-value < 0.05.

## 3. Results

When reporting the results, the experimental groups will be referred to as follows: “Control”: lean control animals receiving standard rat chow and vehicle treatment; “Control+ART”: lean control animals receiving standard rat chow and FDC ART treatment; “HCD”: obese animals receiving standard rat chow + HCD and vehicle treatment; and “HCD+ART”: obese animals receiving standard rat chow + HCD and FDC ART treatment.

### 3.1. The effects of the HCD and ART on biometric, metabolic and oxidative stress parameters

**Fig 2:**
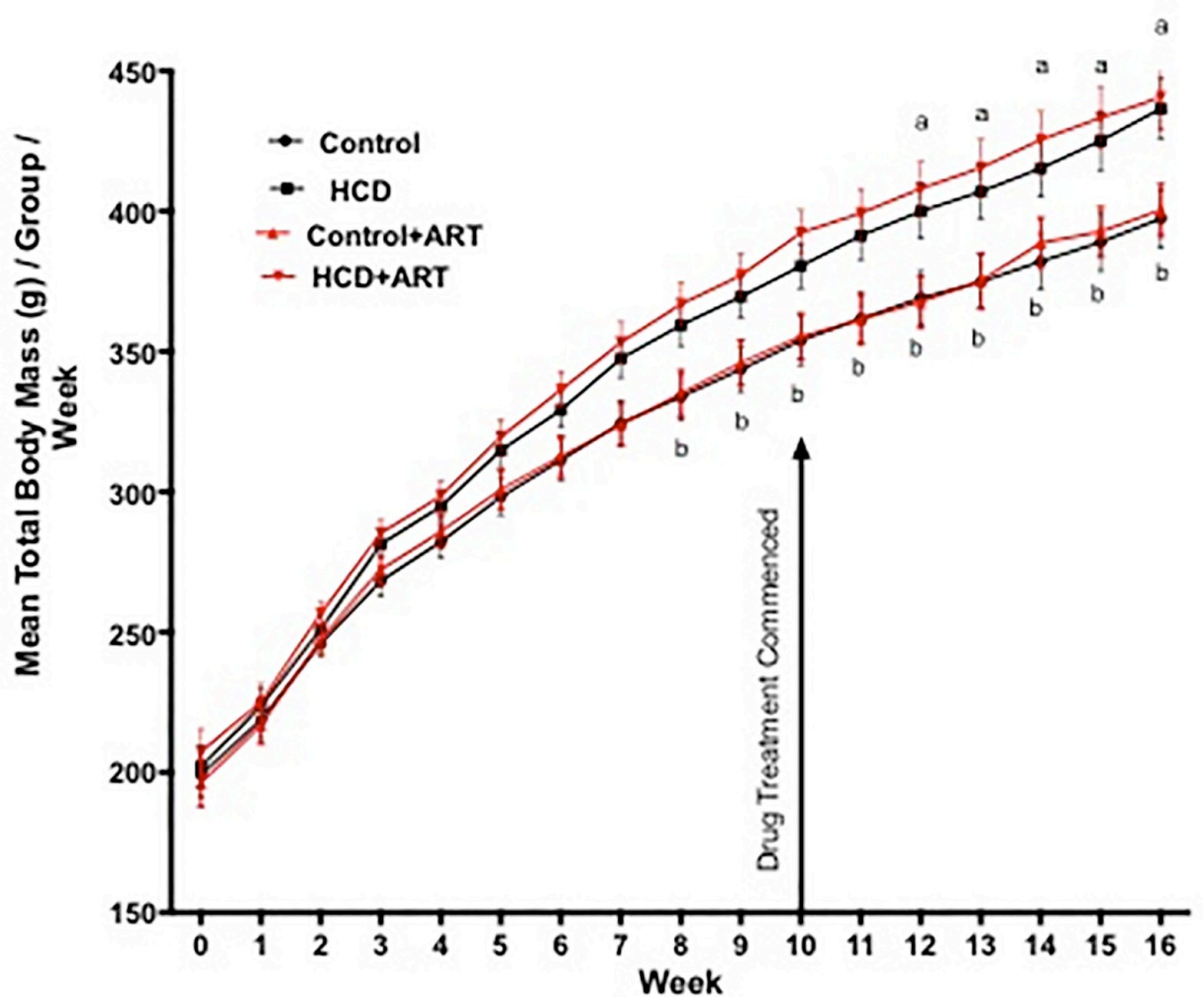
Weekly TBM (g) of the rats in each experimental group expressed as the mean TBM / group during the 16-week feeding programme. Control, *n* = 28; HCD, *n* = 26; Control+ART, *n* = 27; HCD+ART, *n* = 32. ^a^p < 0.05 HCD *vs*. Control ^b^p < 0.05 HCD+ART *vs*. Control+ART. Data analysed by 2-way ANOVA, followed by Bonferroni multiple comparison.

The weekly TBM data are shown in Fig 2. At the end of the 16-week feeding programme, the HCD and HCD+ART rats were ~9.9% and ~10% heavier compared to their respective lean controls; however, ART did not affect body mass measurements in either the control of HCD animals **(Fig 2 and Table 1).**

**Table 1:** Effects of HCD and ART on biometric, metabolic and oxidative stress parameters.

	Control	HCD	Control+ART	HCD+ART
**Final TBM (g)** *(n* = 24–27 / group)	397.4 ± 10.53	436.6 ± 10.68^a^	400.4 ± 9.24	440.7 ± 11.66^b^
**IP Fat Mass (% of TBM)** (*n* = 24–26 / group)	3.61 ± 0.24	5.83 ± 0.29^a^	3.09 ± 0.25	5.91 ± 0.34^b^
**Liver Mass (% of TBM)** (*n* = 24–28 / group)	3.13 ± 0.06	2.93 ± 0.06	3.31 ± 0.08	3.25 ± 0.09^c^
**Heart Mass (% of TBM)** (*n* = 9–11 /group)	0.40 ± 0.01	0.39 ± 0.01	0.40 ± 0.02	0.39 ± 0.01
**Glucose (mmol/L)** (*n* = 5–6 /group)	6.67 ± 0.55	5.82 ± 0.38	5.92 ± 0.57	6.88 ± 0.70
**TC (mmol/L)** (*n* = 4–6 /group)	1.18 ± 0.16	1.11 ± 0.07	1.56 ± 0.14	1.07 ± 0.07^b^
**HDL (mmol/L)** (*n* = 5–6 /group)	0.65 ± 0.05	0.73 ± 0.05	0.58 ± 0.05	0.65 ± 0.08
**TG (mmol/L)** (*n* = 5–6 /group)	0.20 ± 0.04	0.37 ± 0.06^a^	0.22 ± 0.02	0.30 ± 0.02^b^
**CD (μmol/L)** (*n* = 5 /group)	119.5 ± 4.74	136.0 ± 7.76^a^	133.6 ± 15.48	117.9 ± 2.71^c^
**TBARS (μmol/L)** (*n* = 4–6 /group)	20.01 ± 0.71	23.05 ± 0.90^a^	24.63 ± 1.15^a^	22.35 ± 2.38

^a^*p* < 0.05 *vs*. Control

^b^*p* < 0.05 *vs*. Control+ART

^c^*p* < 0.05 *vs*. HCD. Data analysed by 1-way ANOVA (followed by Bonferroni multiple comparison) or student’s t-test.

In addition to the increased TBM, the HCD-induced obese rat model was further characterized by increased % IP fat mass (expressed as absolute IP fat mass / TBM %), as well as increased serum TG and TBARS levels at the end of the 16-week feeding programme compared to lean control animals **(Table 1)**. In the HCD-induced obese rats, treatment with the ART drug resulted in increased % liver mass compared to untreated obese rats; furthermore, ART was associated with reduced serum TC and increased TG levels compared to Control+ART rats, and with reduced CD levels compared to untreated HCD rats **(Table 1)**. In control animals, treatment with the ART drug had no effect on any of the biometric parameters, but did result in increased TBARS levels compared to untreated control **(Table 1)**. Neither HCD-induced obesity nor ART treatment exerted any effects on fasting serum glucose or HDL levels.

### 3.2. The response of hearts to I/R injury: Functional performance and infarct size

#### 3.2.1. Functional performance

There were no differences in any of the pre-I/R functional performance parameters between the groups, suggesting that baseline function (following 30 minutes of stabilisation on the perfusion system) was not affected by any of the experimental interventions **(Table 2)**.

**Table 2:** Functional performance of the hearts pre- and post-global and regional I/R.

				Control	HCD	Control+ART	HCD+ART
**Stabilisation (Pre—I/R)** (*n* = 11–16)			**PSP** (mmHg)	105.13 ± 1.57^a^	100.79 ± 2.40^b^	101.72 ± 1.69	99.15 ± 2.03^c^
			**AO** (ml/min)	42.09 ± 1.51^d^	41.25 ± 1.70^b^	42.36 ± 1.87^e^	38.46 ± 1.57^f^
			**CO** (ml/min)	56.22 ± 1.84^d^	55.21 ± 2.00^b^	57.91 ± 1.81^e^	54.55 ± 2.41^f^
			**Wt** (mW)	13.24 ± 0.52^d^	12.35 ± 0.43^b^	13.25 ± 0.53^e^	12.24 ± 0.61^f^
**Reperfusion** **(Post—I/R)**	**Global I/R** (*n* = 4–6)		**PSP** (mmHg)	83.32 ± 16.79	30.20 ± 19.40^g^	86.00 ± 8.37	66.83 ± 16.79
			**AO** (ml/min)	16.58 ± 5.34	2.10 ± 2.10^g^	14.50 ± 5.85	7.17 ± 4.83
			**CO** (ml/min)	27.33 ± 7.00	6.60 ± 4.21^g^	30.25 ± 9.84	17.92 ± 6.10
			**Wt** (mW)	6.08 ± 1.58	0.94 ± 0.94^g^	6.37 ± 2.28	3.56 ± 1.41
	**Regional I/R** (*n* = 6–10)		**PSP** (mmHg)	72.10 ± 12.11	63.43 ± 13.62	85.83 ± 3.98	77.00 ± 12.99
			**AO** (ml/min)	6.20 ± 2.86	0.57 ± 0.57	9.17 ± 2.02	6.14 ± 3.00^h^
			**CO** (ml/min)	14.60 ± 4.58	9.79 ± 0.71	26.25 ± 2.81^i^	18.50 ± 4.38^h^
			**Wt** (mW)	2.91 ± 0.99	1.40 ± 0.30	4.82 ± 0.49	3.47 ± 1.02^h^
**Hearts Exhibiting Recovery (Producing AO) During Reperfusion**			**Global I/R**	5/6 (83%)	1/5 (20%)	3/4 (75%)	2/6 (33%)
			**Regional I/R**	5/10 (50%)	1/7 (14%)	5/6 (83%)	4/7 (57%)

Pre- vs. Post-I/R

^a^*p* < 0.05 *vs*. Control Post-Regional I/R

^b^*p* < 0.05 *vs*. HCD Post-Global and Regional /R

^c^*p* < 0.05 *vs*. HCD+ART Post-Global I/R

^d^*p* < 0.05 *vs*. Control Post-Global and Regional I/R

^e^*p* < 0.05 *vs*. Control+ART Post-Global and Regional I/R

^f^*p* < 0.05 *vs*. HCD+ART Post-Global and Regional I/R

Post-Global I/R:

^g^*p* < 0.05 *vs*. Control

Post-Regional I/R:

^h^*p* < 0.05 *vs*. HCD

^i^*p* < 0.05 *vs*. Control.

Values obtained from each group before ischaemia were not significantly different and were therefore grouped together. Hearts that did not recover during reperfusion were included in post-I/R statistical analysis. Data analysed by 1-way ANOVA (followed by Bonferroni multiple comparison) or student’s t-test.

After exposure to **global I/R**, hearts from the HCD group showed a significant reduction in mechanical function with respect to all the measured parameters compared to control hearts. In the HCD+ART group, although drug treatment was associated with an improvement in functional parameters during reperfusion in global I/R exposed hearts vs. untreated HCD hearts, these changes did not reach significance. Furthermore, ART had no effect on the functional recovery of control hearts during reperfusion after global ischaemia.

When exposed to the **regional I/R** protocol, functional recovery during reperfusion did not differ between the HCD and control hearts; whereas in HCD+ART hearts, AO, CO and Wt were significantly increased compared to the untreated HCD hearts. Interestingly, the untreated HCD hearts showed the weakest performance in terms of AO (post-regional ischaemia) compared to the untreated control and HCD+ART groups. Control+ART hearts showed an increased CO compared to the untreated control group. The improvement in post-regional I/R function in the HCD+ART hearts was further underscored by the increase in the number of hearts that recovered sufficiently to generate AO during reperfusion: from only 14% of the hearts in the untreated HCD group to 57% in the HCD+ART group. Interestingly, ART treatment also increased the number of AO-generating control hearts from 50% to 83% **(Table 2)**.

#### 3.2.2. Infarct size development

**Fig 3:**
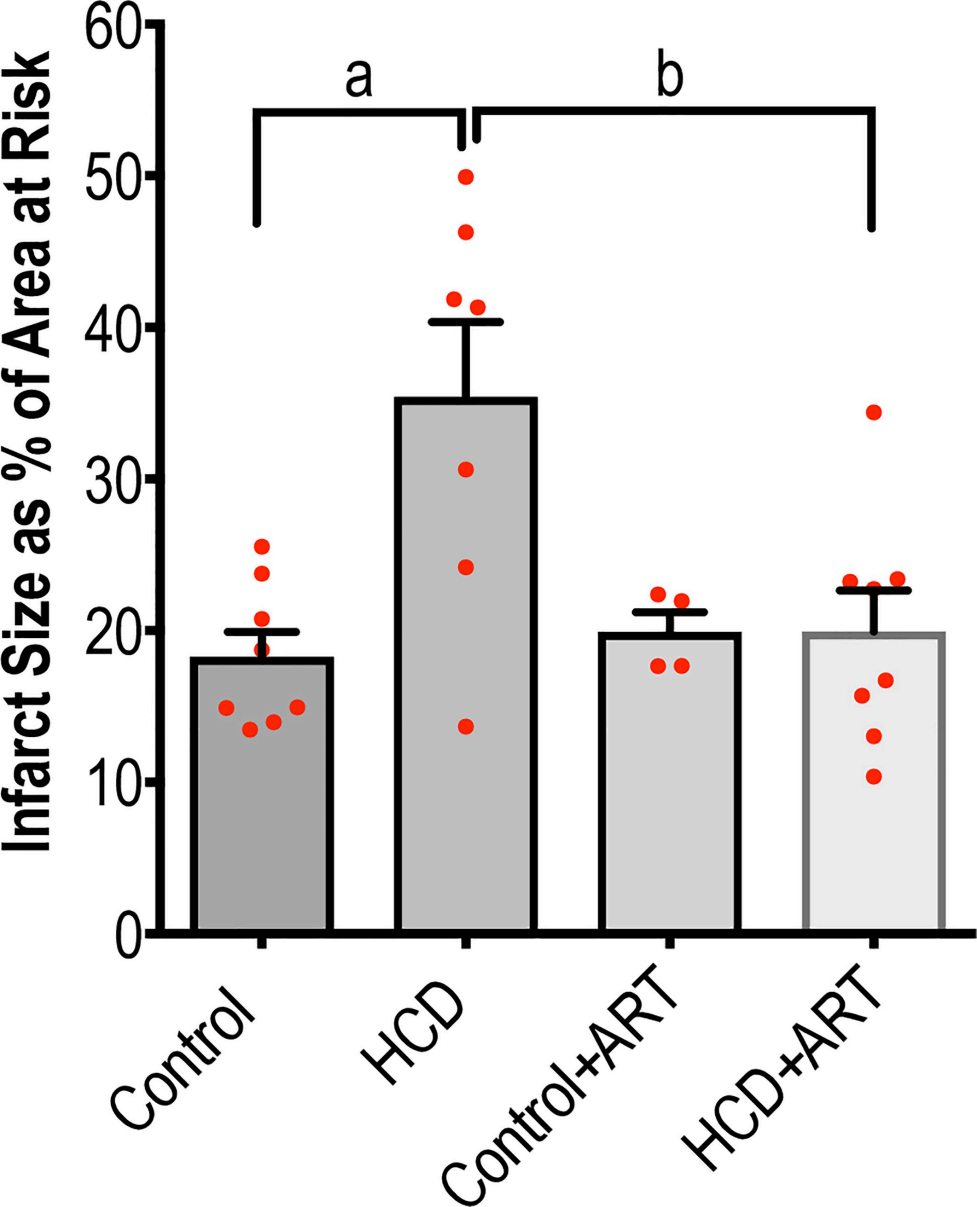
IS expressed as percentage of the area at risk. Control: 18.26 ± 1.66; HCD: 35.40 ± 4.95; Control+ART: 19.91 ± 1.30; HCD+ART: 19.95 ± 2.70. Control, *n* = 8; HCD, *n* = 7; Control+ART, *n* = 4; HCD+ART, *n* = 8. ^a^*p* < 0.05, ^b^*p* < 0.05. Data analysed by 1-way ANOVA (followed by Bonferroni multiple comparison).

Myocardial infarction was induced in the hearts by ligation of the left anterior descending coronary artery, after which the size of the infarcts was measured. In hearts from the HCD-induced obese rats, the IS was significantly larger compared to control hearts. The increase in IS was reversed in HCD hearts treated with ART **(Fig 3)**, further supporting the beneficial effects exerted by ART on the mechanical function of HCD hearts exposed to regional ischaemia. No significant inter-group differences were observed in the % area at risk (Control: 40.16 ± 2.99%; HCD: 31.61 ± 2.29%; Control+ART: 38.91 ± 2.13%; HCD+ART 39.53 ± 4.80%; *p* > 0.05), which validated that the LAD ligation procedure was consistent in all groups and that the differences in IS were not a result of technical reasons.

### 3.3. Baseline and post-ischaemic expression and activation by phosphorylation of proteins involved with nitric oxide (NO) production (eNOS), cardioprotection (PKB/Akt), metabolic regulation (AMPK), and oxidative stress (nitrotyrosine and p22 phox) in hearts from rats exposed to HCD and ART

#### 3.3.1. eNOS

**Fig 4:**
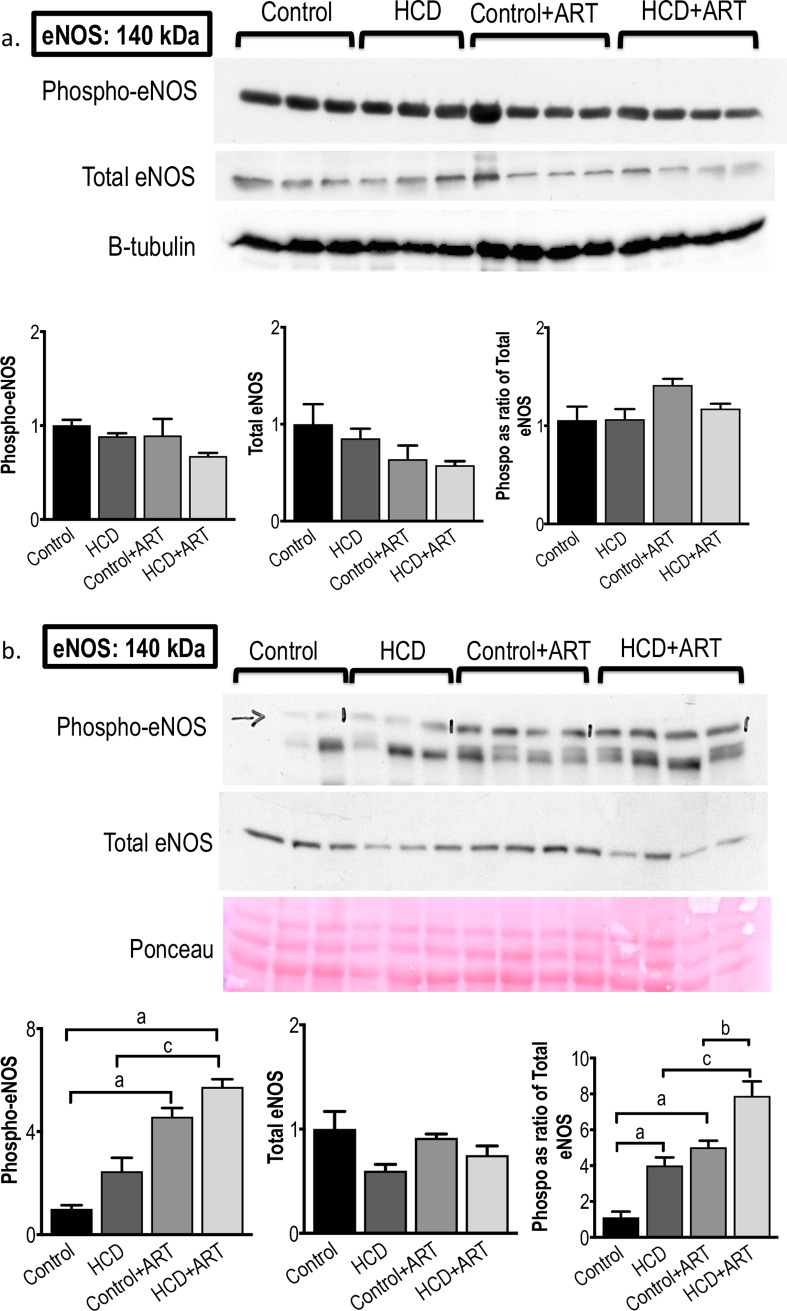
**Western blot measurements of eNOS protein expression and phosphorylation at (a) baseline conditions, and (b) post-global I/R conditions.** *n* = 3–4 /group. ^a^*p* < 0.05 *vs*. Control, ^b^*p* < 0.05 *vs*. Control+ART, ^c^*p* < 0.05 *vs*. HCD. Data analysed by 1-way ANOVA (followed by Tukey’s multiple comparison).

Under baseline conditions, total myocardial eNOS protein and phosphorylated (activated) eNOS levels were not affected by any of the experimental interventions, although there was a trend (*p* = 0.06) for the phosphorylated / total eNOS ratio to increase by ~33% in control+ART hearts *vs*. untreated control **(Fig 4A)**. Exposure to 20 min global ischaemia / 60 min reperfusion resulted in ~5.7-fold increase in phosphorylated eNOS in HCD+ART hearts *vs*. untreated control and ~2.3-fold *vs*. untreated HCD. The I/R protocol also increased phosphorylated eNOS levels by ~4.6-fold in control+ART *vs*. untreated control. Total eNOS expression remained unchanged, and the phosphorylated / total eNOS ratios were increased in HCD+ART hearts (~7.1-fold *vs*. untreated control, ~2-fold *vs*. untreated HCD and ~1.6-fold *vs*. control+ART), in HCD hearts (~3.6-fold *vs*. untreated control), and in control+ART hearts (~4.5-fold *vs*. untreated control) **(Fig 4B)**.

#### 3.3.2. PKB / Akt

**Fig 5:**
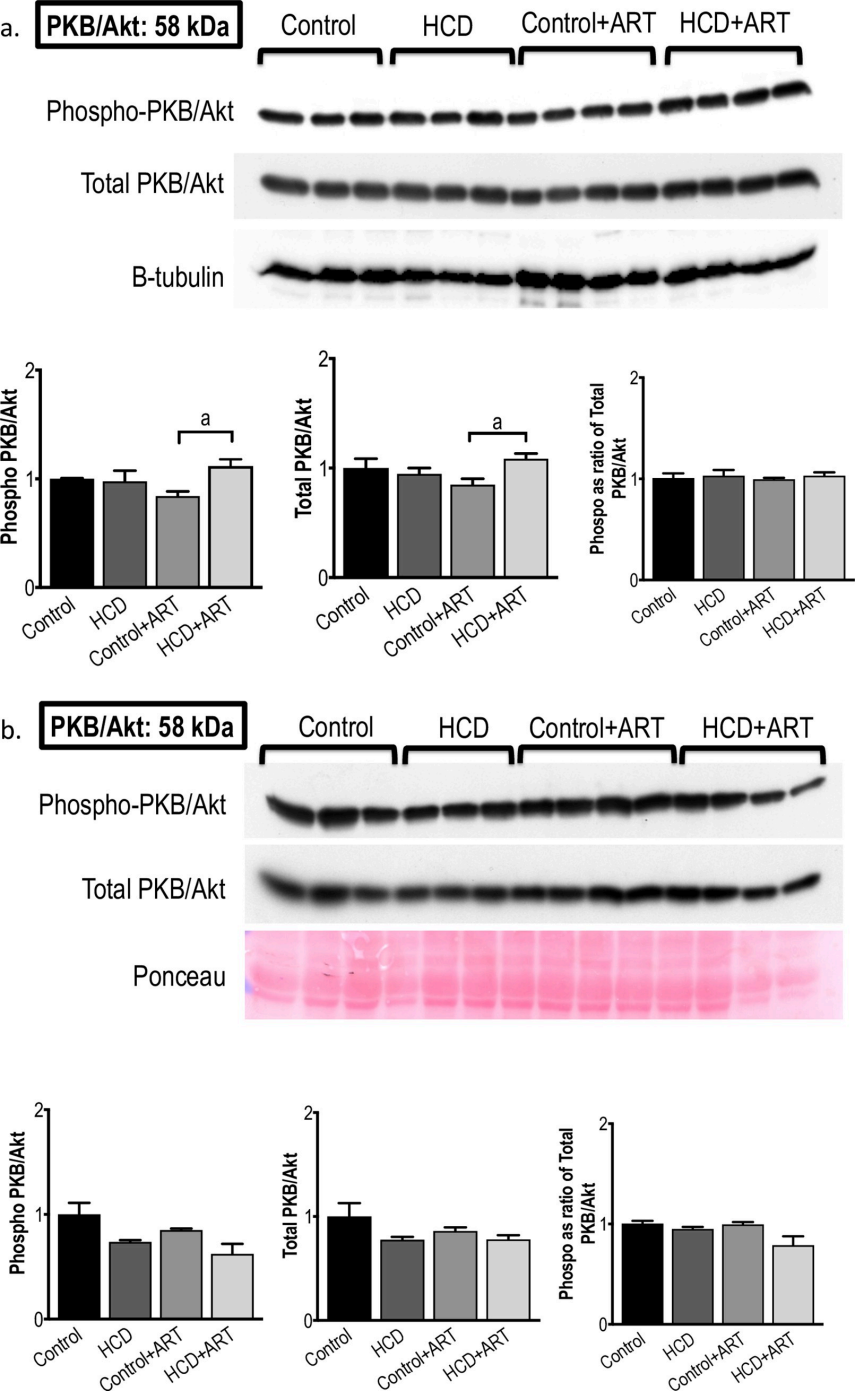
**Western blot measurements of PKB / Akt protein expression and phosphorylation at (a) baseline conditions, and (b) post-global I/R conditions.** *n* = 3–4 /group. ^a^*p* < 0.05 *vs*. Control+ART. Data analysed by 1-way ANOVA (followed by Tukey’s multiple comparison).

In baseline hearts, the total and phosphorylated (activated) PKB / Akt levels were lower in control+ART hearts compared to HCD+ART hearts; however, no differences were observed in the phosphorylated / total PKB / Akt ratios between any of the groups **(Fig 5A)**. In hearts exposed to global I/R, phosphorylated PKB / Akt levels were significantly decreased in the HCD+ART group (~38% *vs*. untreated control); however total protein levels and phosphorylated / total ratios remained similar in all groups **(Fig 5B)**.

#### 3.3.3. AMPK

**Fig 6:**
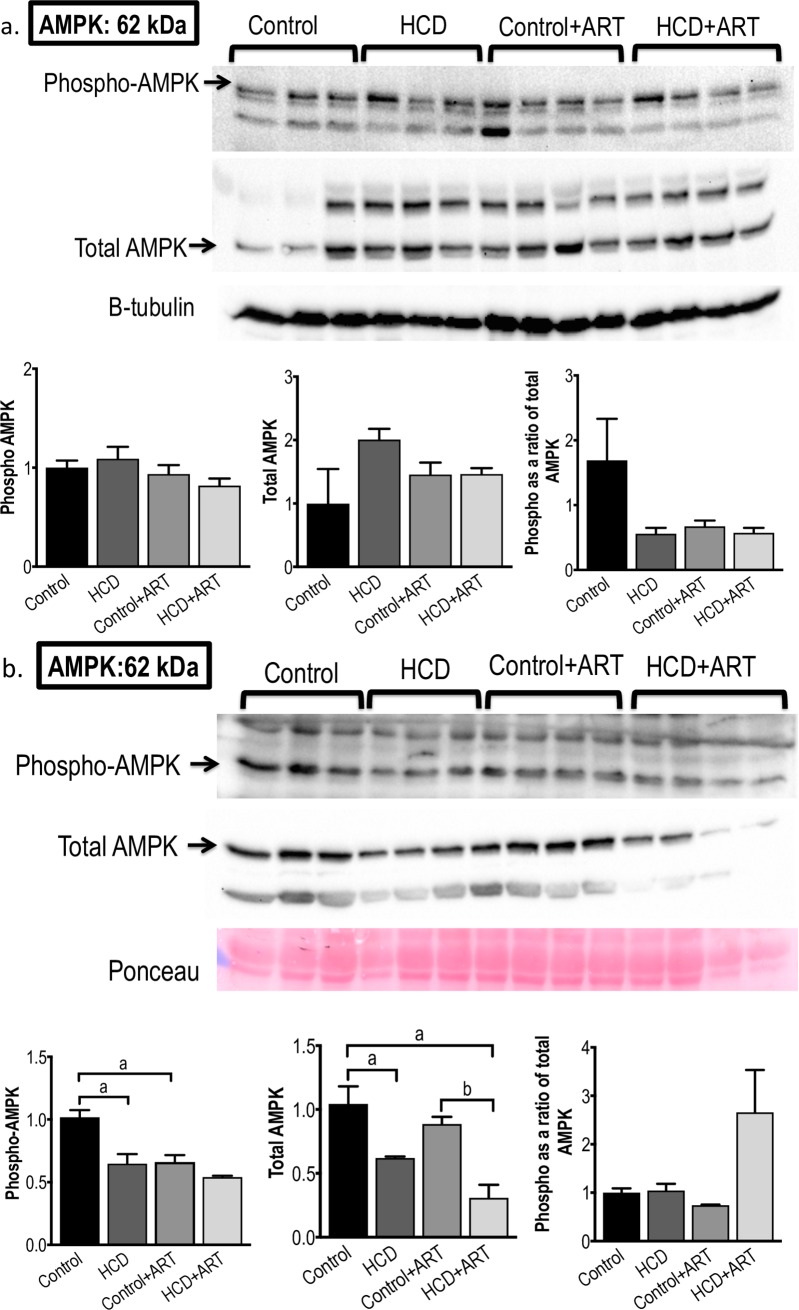
**Western blot measurements of AMPK protein expression and phosphorylation at (a) baseline conditions, and (b) post-global I/R conditions.** *n* = 3–4 /group. ^a^*p* < 0.05 *vs*. Control, ^b^*p* < 0.05 *vs*. Control+ART. Data analysed by 1-way ANOVA (followed by Tukey’s multiple comparison).

Under baseline conditions, none of the experimental interventions exerted any significant effects on myocardial AMPK levels **(Fig 6A)**. However, after exposure to global I/R, phosphorylated AMPK levels were decreased by ~50% in HCD+ART *vs*. control, ~40% in HCD *vs*. control, and ~33% in control+ART *vs*. control. In addition, total AMPK protein levels were ~68% decreased in HCD+ART *vs*. control, ~59% decreased in HCD+ART *vs*. control+ART, and ~40% decreased in HCD *vs*. control,. No changes were observed in the phosphorylated / total AMPK ratios **(Fig 6B)**.

#### 3.3.4. Nitrotyrosine

**Fig 7:**
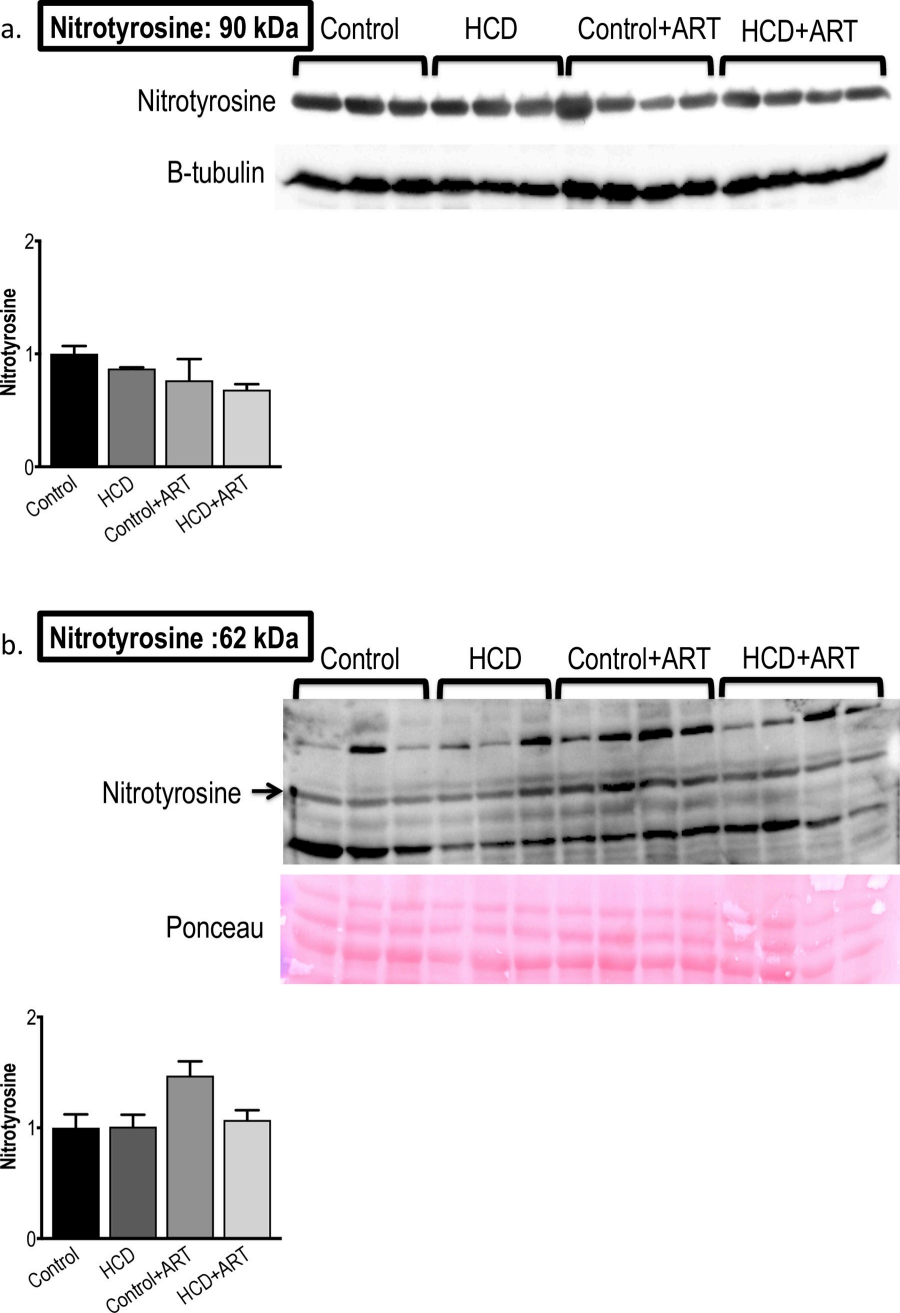
**Western blot measurements of nytrotyrosine protein expression at (a) baseline conditions, and (b) post-global I/R conditions.** *n* = 3–4 /group. Data analysed by 1-way ANOVA (followed by Tukey’s multiple comparison).

Myocardial nitrotyrosine levels were not affected by any of the experimental interventions under baseline conditions **(Fig 7A)**. After global ischaemia, no inter-group differences were observed, except in control+ART hearts, where nitrotyrosine levels trended to be higher compared to untreated control (~47% increase; *p* = 0.06) **(Fig 7B)**.

#### 3.3.5. p22 phox

**Fig 8:**
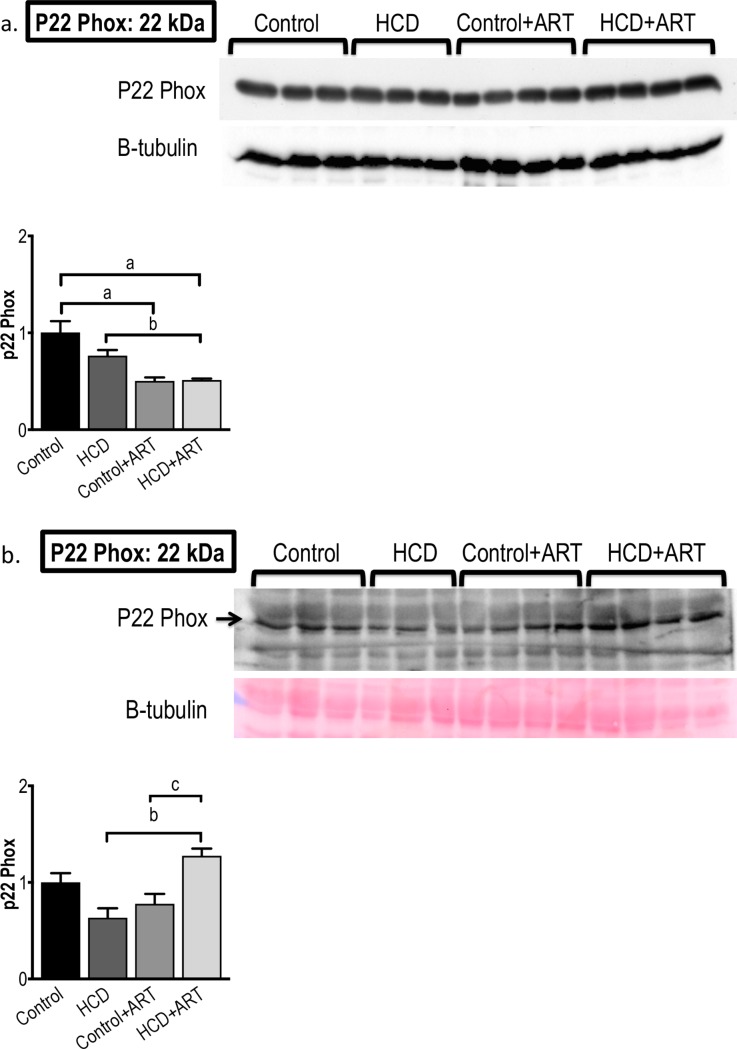
**Western blot measurements of p22 phox protein expression at (a) baseline conditions, and (b) post-global I/R conditions.** *n* = 3–4 /group. ^a^*p* < 0.05 *vs*. Control, ^b^*p* < 0.05 *vs*. HCD, ^c^*p* < 0.05 *vs*. Control+ART. Data analysed by 1-way ANOVA (followed by Tukey’s multiple comparison).

Under baseline conditions, p22 phox levels in HCD+ART hearts were ~25% decreased *vs*. untreated HCD, and ~40% decreased *vs*. untreated control. Levels were also ~50% decreased in control+ART hearts *vs*. control **(Fig 8A)**. After global ischaemia, p22 phox levels were ~2-fold higher in HCD+ART hearts compared to untreated HCD, and ~1.6-fold higher *vs*. control+ART **(Fig 8B)**.

## 4. Discussion

In the present study, we investigated the cardiac and metabolic effects of a commercially available FDC ART drug, containing a combination of two NRTI’s (TDF and FTC) and one NNRTI (EFV), in a rat model of diet-induced obesity. The rationale for the study arises from the now well recognized increased risk of myocardial infarction in HIV-infected populations, together with increasing obesity rates that are often observed at the time of HIV diagnosis before ART initiation [11]. Obesity, a modifiable cardiovascular risk factor, could potentially pose an additional risk for ischaemic heart disease in HIV-infected individuals. Furthermore, the specific drug combination under investigation in this study, is one that is strongly recommended by the WHO for first line ART [16]. Combination ART drugs are recommended in view of the ability of the HI-virus to develop drug resistance against individual drugs used as mono-therapy. Notably, this FDC ART drug does not contain a PI, which is relatively well researched, and has previously been shown to be associated with increased myocardial infarction risk and metabolic derangements [39,40]. On the other hand, the role of the TDF, FTC and EFV combination drug in hearts from obese subjects, especially in the context of I/R injury, is not well described, and it is not known whether the susceptibility of the hearts to I/R injury is affected by the drug. In view of the above knowledge gaps, we investigated the cardiac and metabolic effects of a recently introduced FDC ART drug (consisting of TDF, FTC and EFV; commercially available as Odimune) administered once daily to HCD-induced obese rats for six weeks, comparing the findings with appropriate lean and vehicle treated control animals.

The main findings of the study are (1) the FDC ART treatment did not alter the obese phenotype of the rats in terms of biometric or metabolic variables; however, serum CD levels (marker of lipid peroxidation) were decreased with ART, (2) HCD-induced obesity resulted in weaker cardiac function and larger IS in hearts subjected to a simulated myocardial infarction protocol and I/R injury; in a novel finding, treating the obese rats with the FDC ART protected their hearts against the ischaemic insult, as evidenced by improved post-I/R functional performance and reduced IS, (3) measurements of cardiac proteins revealed that the ART-induced cardioprotection was associated with increased activation of the cardioprotective eNOS protein and reduced activation of AMPK, an important regulatory protein of energy production in the heart. (4) overall, the protection conferred by the FDC ART in the obese hearts against I/R injury appeared to be associated with direct cardiac mechanisms, most notably the up-regulation of eNOS, rather than an improvement in the biometric and metabolic profile of the animals.

### 4.1. The biometric and metabolic effects of the FDC ART drug treatment

At the end of the 16-week feeding programme, the HCD-induced obesity phenotype was characterised by increased TBM, increased visceral fat mass, as well as increased serum TG, CD and TBARS levels compared to control rats **(Table 1)**, as shown by others using similar HCD’s [36,41–43]. Fasting blood glucose levels were not affected, suggesting that insulin resistance and diabetes were not induced by the feeding programme, as previously shown by others using similar diets [44,45]. Our data show that body and IP fat weights were not altered by the FDC ART treatment in either the obese or control animals. However, ART-treated obese rats did present with increased liver: body mass ratios **(Table 1)**, suggesting that ART may have induced hepatomegaly in the obese animals. NRTIs and PIs are implicated in the pathogenesis of HIV-related lipodystrophy, and NRTIs in particular are associated with cardiac and hepatic lipid accumulation, and hepatomegaly [46]. Overall, our biometric data suggest that the FDC ART regimen did not induce lipodystrophy-like effects in either the control or HCD-induced obese rats, with the exception of modestly increased liver weights in the ART exposed obese group.

In our hands, the elevated TG levels observed in the untreated obese animals remained high in the FDC ART exposed animals, whereas the ART-exposed obese rats had lower TC levels compared to ART-treated control rats, suggesting that the drug exerted favourable effects on TC levels in the presence of obesity **(Table 1)**. In the non-obese control rats, the TC, HDL and TG levels were unaffected by the FDC ART. We are not aware of previous studies investigating the effects of this FDC ART on blood lipids in the context of obesity. In the non-obese setting, ART has previously been associated with dyslipidaemia [47–49] and hypercholesterolemia [50–52] with PIs seemingly the main culprits. Mono-therapy with TDF, one of the components of the current study’s combination drug, has previously been shown to exert favourable effects on blood lipids in a clinical study [53]; whereas another FDC ART component, EFV, has been associated with dyslipidaemia [54]. In the present study, treatment with the FDC ART did not affect fasting glucose levels in either control or HCD rats. As far as we are aware, no data are available on the effects of this specific FDC drug on blood glucose levels in obese animals. However, in the non-obese context, ART has been associated with impaired glucose homeostasis [55,56]. On the other hand, a study in ART exposed HIV-infected participants failed to demonstrate changes in fasting blood glucose, despite observing a positive association between ART and obesity [57]. In summary, the FDC ART did not significantly alter the metabolic profile of the obese rats, with the exception of reduced TC levels when compared to treated control rats.

Serum markers of lipid peroxidation were differentially affected by treatment with the FDC ART drug in the control and obese groups. TBARS levels were increased in ART-exposed control animals; whereas CD levels were lower in ART-exposed obese animals compared to the control+ART group **(Table 1)**, suggesting that the combination drug exerted favourable effects on CD levels in the obese, but not in the lean, animals. More studies are required to investigate this phenomenon. Despite the lack of studies investigating oxidative stress in the context of FDC ART and obesity, it is known that HIV-infected ART exposed individuals are at risk of developing systemic oxidative stress [40], due to the failure of anti-oxidant systems and/or increased ROS production [58]. Mono-therapy with EFV, one of the components of the current study’s FDC ART drug, has previously been shown to result in higher levels of systemic oxidative stress markers compared to a PI-based regimen in HIV-infected participants [59]; in contrast, another study showed that NNRTI-treated patients had lower oxidative stress levels [60]. Overall, the experimental design of the current study did not include groups treated with the individual components of the FDC ART drug, which precludes comprehensive comparative insights, but it appears that the combination drug may have a favourable effect in the obese rats.

### 4.2. The cardiac effects of the FDC ART drug treatment

HIV-associated cardiomyopathy (particularly common in the pre-highly active ART (HAART) era) still persists today, albeit at lower rates [61]. For example, in a study conducted in 2011, ART exposed persons showed an unexpectedly high prevalence of left ventricular dysfunction and hypertrophy; in addition, it was shown that cardiomyopathy was independently associated with increased body weight and the use of an NRTI [62]. In our hands, using an *ex vivo* working rat heart model, the FDC ART drug did not affect any of the baseline myocardial function parameters in either control or HCD rat hearts, thus excluding the development of myocardial dysfunction/cardiomyopathy **(Table 2)**. We are not aware of any previous animal studies that investigated the effects of this particular FDC ART drug on cardiac function.

Although baseline myocardial functional performance was not affected, HCD-induced obesity was associated with reduced myocardial functional performance and increased infarct development after exposure to I/R injury **(Table 2; Fig 3)**, as previously shown by others [63–65]. These findings confirm that the diet-induced obesity model of the current study negatively impacted on the hearts’ susceptibility to a simulated myocardial infarction procedure and I/R protocol. Our results furthermore show that the FDC ART treatment was overall cardioprotective. Hearts from ART-treated control rats exposed to regional ischaemia generated higher cardiac outputs during reperfusion *vs*. untreated control hearts. The improved post-ischaemic function was even more evident in the hearts from the ART-treated obese rats, showing significant improvements in AO, CO and Wt **(Table 2)**. Limited data are available on the effects of NRTIs and NNRTIs in hearts exposed to I/R injury. As far as we are aware, no data are available on pre- and post-ischaemic cardiac functional performance in the context of ART in general, and the FDC ART drug used in the present study in particular, thus rendering our data novel. The effects of treatment with the FDC ART drug on infarct development were also determined following a regional I/R procedure that involved ligation of the left anterior descending coronary artery. Results showed that the infarcts were significantly smaller, which underscores the improved functional performance observed in the FDC ART exposed HCD hearts **(Fig 3)**. These findings are novel, and provide evidence that the particular FDC ART drug used in the current study, reduces the susceptibility of hearts from obese rats to myocardial infarction and I/R injury. While clinical evidence is relatively conclusive regarding the myocardial infarction risk of PIs, more data are required on the effects of NRTIs and NNRTIs [66]. Interestingly, a large cohort study reported that exposure to two of the components of the FDC drug used in the present study, TDF and EFV, were not associated with increased risk of myocardial infarction compared to the PI, lopinavir-ritonavir [3].

To gain further information on the putative direct cardiac mechanisms of the FDC ART drug, the expression and activation of several cardiac proteins were measured. Significant increases were observed in absolute phosphorylated eNOS levels and phosphorylated / total eNOS ratios in FDC ART-treated control and obese rat hearts subjected to I/R **(Fig 4)**. In view of the known cardioprotective properties of cardiac eNOS-NO [67], we postulate that the strong post-ischaemia increases in eNOS activation may, at least in part, be a candidate mechanism underlying the ART-associated cardioprotection observed in both functional recovery and IS development. We are not aware of any other studies that investigated myocardial eNOS in the context of ART, and certainly not in hearts exposed to I/R. Future studies exploring ART-induced eNOS activation patterns and the effects of manipulation (genetic or pharmacological) of the eNOS pathway may shed more light as to the exact role of eNOS in myocardial protection by this particular FDC ART drug.

Although ART resulted in higher baseline levels of phosphorylated PKB/Akt in HCD hearts compared to control+ART hearts, there were no differences in the phosphorylated/total PKB/Akt ratios **(Fig 5)**. In the post-I/R scenario, phosphorylated PKB/Akt levels were significantly decreased in the FDC ART exposed obese hearts compared to untreated control; however, the significance was lost when data were expressed as ratios of total protein. The PKB/Akt pathway has previously been shown to be targeted by ART; for example, HAART-treated HIV-infected participants with signs of lipodystrophy showed impaired insulin signalling in skeletal muscle at the level of PKB/Akt [68]. We are not aware of any animal studies investigating myocardial PKB/Akt in the context of I/R injury in ART and obesity. It is therefore difficult to place our results in context; however, overall the rather modest changes observed in PKB/Akt following FDC ART-treatment suggest that this pro-survival protein is an unlikely candidate mechanism of the protection observed in the post-I/R HCD-induced obese hearts. In the present study, treatment with the FDC ART drug did not affect baseline myocardial AMPK levels. However, in the post-I/R setting, total and phosphorylated AMPK levels were significantly lower in ART-treated control and obese groups, which coincided with the cardioprotection elicited by the treatment, suggesting a lesser degree of metabolic stress in these hearts **(Fig 6)**, in view of AMPK’s role as a major regulator of energy production [26].

In baseline hearts, nitrotyrosine levels remained unchanged in all groups; however, p22 phox levels were lower in the ART-treated control and HCD groups, suggesting that, under baseline conditions, the FDC ART may have conferred inhibition of myocardial NADPH-oxidase activity and hence possibly reduced reactive oxygen species (ROS) levels **(Figs 7 and 8)**. In contrast to our findings with the FDC ART drug, a previous study in rats exposed to mono-therapy with a single NRTI, showed increased myocardial NADPH-oxidase activity [69]. Increased myocardial nitrotyrosine levels were shown in NRTI / PI exposed transgenic HIV mice (18 weeks treatment), which was associated with cardiac dysfunction; however these observations may be the result of synergism between HIV-infection and ART, and the presence of a PI may have played a role [70]. After exposure to I/R, ART-treated control hearts showed a trend towards increased nitrosative stress (increased nitrotyrosine expression; *p* = 0.06); however, no changes were observed in the ART exposed obese group. Furthermore, I/R injury elicited increases in p22 phox levels in hearts from ART-treated obese rats, suggesting that the I/R insult induced an up-regulation of NADPH-oxidase and possibly increased superoxide generation. However, the magnitude of the ART-induced increases in p22 phox levels was seemingly insufficient to result in poorer functional performance or infarct development.

## 5. Conclusion

We have shown that a TDF/FTC/EFV fixed-dose combination ART regimen of 6 weeks’ duration in diet-induced obese rats exerted predominantly favourable effects on metabolic and cardiac endpoints. These findings are novel, and suggest that, in our hands, this particular antiretroviral drug combination does not induce harmful cardiometabolic effects as seen with other ART classes such as PIs. The HCD-induced obese rats particularly benefited from the FDC ART treatment, particularly in terms of their hearts’ responses to simulated myocardial infarction and I/R injury. In a novel finding, the FDC ART drug was associated with significant cardioprotection in I/R exposed hearts from the obese rats, which was underscored by strong up-regulation of myocardial phospho-activated eNOS levels. The findings suggest that the cardioprotection may have been a result of direct cardiac mechanisms rather than an improved metabolic status. Our results raise the possibility that this combination drug may be considered as a possible treatment option in obese HIV-infected individuals with a high risk of developing ischaemic heart disease; however, more research is necessary to ascertain whether the favourable effects are also observed in the presence of HIV-infection.

## 6. Limitations of the study

The study has several shortcomings. Our animal model was HIV-free, which precluded an opportunity to measure the drug effects in the presence of HIV-infection and an inflammatory milieu. On the other hand, using a HIV-free model allowed the researchers to focus on the drug effects *per se*. Future studies in for example murine HIV models or transgenic AIDS mice may provide clarity on how the ART drug affects the cardiometabolic endpoints in the presence of possible viral actions. It may be argued that the ART treatment period of 6 weeks was too short; indeed, a longer treatment period may have provided more explicit findings. We also did not investigate *ex vivo* effects of the drugs administered directly to the hearts during the perfusion protocol. The advantages of such supplementary studies would be to confirm that the cardioprotective effects were direct drug effects and not related to other unknown circulating factors. Finally, this study did not include experimental groups that received the individual components of the combination drug separately. Although this would have added considerably to the complexity of the study with three extra groups of rats, it may have provided clues as to whether the findings were a result of combined effects, or due to the actions of one or more of the individual components.

## Supporting information

S1 Fig Western blot measurements of eNOS protein expression at baseline conditions. *n* = 3–4 /group. (Antibody: polyclonal; source: rabbit; dilution 1:1000)
(PDF)

S2 Fig Western blot measurements of eNOS protein phosphorylation at baseline conditions. *n* = 3–4 /group. (Antibody: polyclonal; source: rabbit; dilution 1:1000)
(PDF)

S3 Fig β-tubulin for Western blot measurements of eNOS at baseline conditions. *n* = 3–4 /group. (Antibody: polyclonal; source: rabbit; dilution 1:1000)
(PDF)

S4 Fig Western blot measurements of eNOS protein expression at post-global I/R conditions. *n* = 3–4 /group. (Antibody: polyclonal; source: rabbit; dilution 1:1000)
(PDF)

S5 Fig Western blot measurements of eNOS protein phosphorylation at post-global I/R conditions. *n* = 3–4 /group. (Antibody: polyclonal; source: rabbit; dilution 1:1000)
(PDF)

S6 Fig Ponceau for Western blot measurements of eNOS at post-I/R conditions. *n* = 3–4 /group.
(PDF)

S7 Fig Western blot measurements of PKB/Akt protein expression at baseline conditions. *n* = 3–4 /group. (Antibody: polyclonal; source: rabbit; dilution 1:1000)
(PDF)

S8 Fig Western blot measurements of PKB/Akt protein phosphorylation at baseline conditions. *n* = 3–4 /group. (Antibody: monoclonal; source: rabbit; dilution 1:1000)
(PDF)

S9 Fig β-tubulin for Western blot measurements of PKB/Akt at baseline conditions. *n* = 3–4 /group. (Antibody: polyclonal; source: rabbit; dilution 1:1000)
(PDF)

S10 Fig Western blot measurements of PKB/Akt protein expression at post-global I/R conditions. *n* = 3–4 /group. (Antibody: polyclonal; source: rabbit; dilution 1:1000)
(PDF)

S11 Fig Western blot measurements of PKB/Akt protein phosphorylation at post-global I/R conditions. *n* = 3–4 /group. (Antibody: monoclonal; source: rabbit; dilution 1:1000)
(PDF)

S12 Fig Ponceau for Western blot measurements of PKB/Akt at post-I/R conditions. *n* = 3–4 /group.
(PDF)

S13 Fig Western blot measurements of AMPK protein expression at baseline conditions. *n* = 3–4 /group. (Antibody: polyclonal; source: rabbit; dilution 1:1000)
(PDF)

S14 Fig Western blot measurements of AMPK protein phosphorylation at baseline conditions. *n* = 3–4 /group. (Antibody: monoclonal; source: rabbit; dilution 1:1000)
(PDF)

S15 Fig β-tubulin for Western blot measurements of AMPK at baseline conditions. *n* = 3–4 /group. (Antibody: polyclonal; source: rabbit; dilution 1:1000)
(PDF)

S16 Fig Western blot measurements of AMPK protein expression at post-global I/R conditions. *n* = 3–4. (Antibody: polyclonal; source: rabbit; dilution 1:1000)
(PDF)

S17 Fig Western blot measurements of AMPK protein phosphorylation at post-global I/R conditions. *n* = 3–4 /group. (Antibody: monoclonal; source: rabbit; dilution 1:1000)
(PDF)

S18 Fig Ponceau for Western blot measurements of AMPK at post-I/R conditions. *n* = 3–4 /group.
(PDF)

S19 Fig Western blot measurements of nytrotyrosine protein expression at baseline conditions. *n* = 3–4 /group. (Antibody: polyclonal; source: rabbit; dilution used 1:200)
(PDF)

S20 Fig β-tubulin for Western blot measurements of nytrotyrosine at baseline conditions. *n* = 3–4 /group. (Antibody: polyclonal; source: rabbit; dilution 1:1000)
(PDF)

S21 Fig Western blot measurements of nytrotyrosine protein expression at post-global I/R conditions. *n* = 3–4 /group. (Antibody: polyclonal; source: rabbit; dilution 1:200)
(PDF)

S22 Fig Ponceau for Western blot measurements of nytrotyrosine at post-I/R conditions. *n* = 3–4 /group.
(PDF)

S23 Fig Western blot measurements of p22 phox protein expression at baseline conditions. *n* = 3–4 /group. (Antibody: polyclonal; source: rabbit; dilution 1:200)
(PDF)

S24 Fig β-tubulin for Western blot measurements of p22 phox at baseline conditions. *n* = 3–4 /group. (Antibody: polyclonal; source: rabbit; dilution 1:1000)
(PDF)

S25 Fig Western blot measurements of p22 phox protein expression at post-global I/R conditions. *n* = 3–4. (Antibody: polyclonal; source: rabbit; dilution 1:200)
(PDF)

S26 Fig Ponceau for Western blot measurements of p22 phox at post-I/R conditions. *n* = 3–4 /group.
(PDF)
