# Supplementary figures and images for: Treatment with a fixed dose combination antiretroviral therapy drug containing tenofovir, emtricitabine and efavirenz is associated with cardioprotection in high calorie diet-induced obese rats
Source: PLoS One. 2018 Dec 5;13(12):e0208537. doi: 10.1371/journal.pone.0208537 (PMC6281242; doi:10.1371/journal.pone.0208537)

eNOS: 140 kDa

Control      HCD      Control+ART      HCD+ART

Total eNOS

Total eNOS →

CONTROL      HF      C-ART      HF-ART

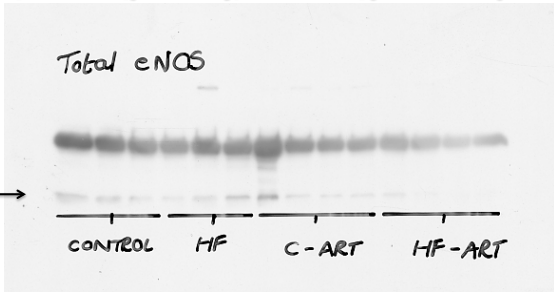

Supplement: S1 Fig — n = 3–4 /group. (Antibody: polyclonal; source: rabbit; dilution 1:1000) (PDF) [file pone.0208537.s001.pdf]

eNOS: 140 kDa

Control      HCD      Control+ART      HCD+ART

p eNOS

Phospho-eNOS →

CONTROL      HF      C-ART      HF-ART

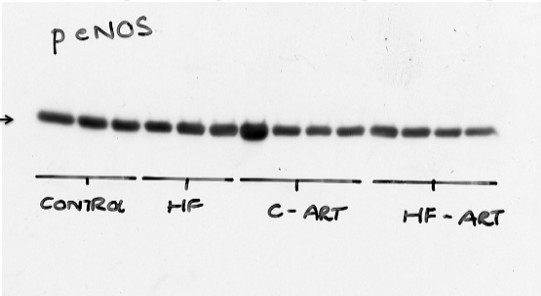

Supplement: S2 Fig — n = 3–4 /group. (Antibody: polyclonal; source: rabbit; dilution 1:1000) (PDF) [file pone.0208537.s002.pdf]

**eNOS: 140 kDa**

Control

HCD

Control+ART

HCD+ART

B-tubulin →

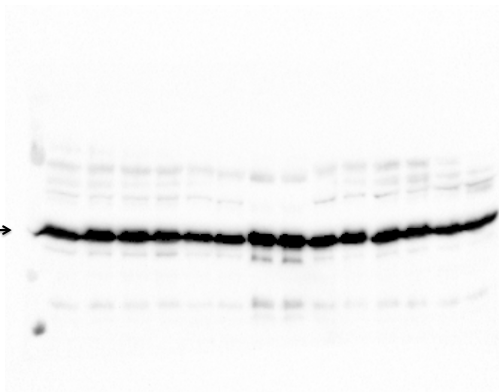

Supplement: S3 Fig — n = 3–4 /group. (Antibody: polyclonal; source: rabbit; dilution 1:1000) (PDF) [file pone.0208537.s003.pdf]

eNOS: 140 kDa

Control

HCD

Control+ART

HCD+ART

Phospho eNOS : Lean vs. obese (+/- ART)

Phospho-eNOS →

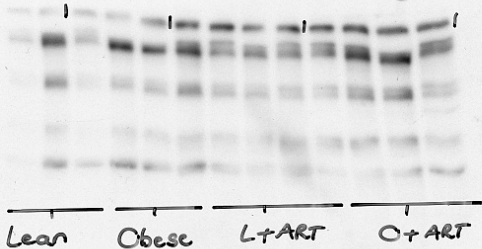

Supplement: S5 Fig — n = 3–4 /group. (Antibody: polyclonal; source: rabbit; dilution 1:1000) (PDF) [file pone.0208537.s005.pdf]

**eNOS: 140 kDa**

Control      HCD      Control+ART      HCD+ART

Ponceau →

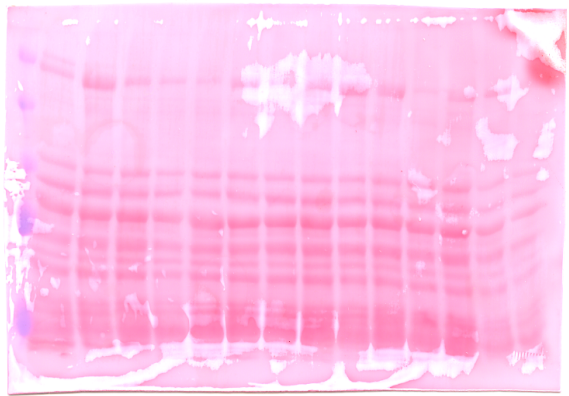

Supplement: S6 Fig — n = 3–4 /group. (PDF) [file pone.0208537.s006.pdf]

PKB/Akt: 58 kDa

Control

HCD

Control+ART

HCD+ART

TOTAL PKB (NEW ab)

26.Cx6.14

Total PKB/Akt →

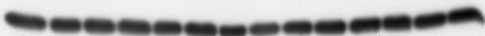

CONTROL

HF

C-ART

HF-ART

Supplement: S7 Fig — n = 3–4 /group. (Antibody: polyclonal; source: rabbit; dilution 1:1000) (PDF) [file pone.0208537.s007.pdf]

PKB/Akt: 58 kDa

Control

HCD

Control+ART

HCD+ART

Phospho PKB

Phospho  
PKB/Akt →

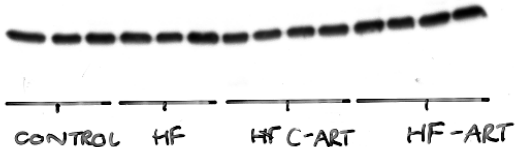

Supplement: S8 Fig — n = 3–4 /group. (Antibody: monoclonal; source: rabbit; dilution 1:1000) (PDF) [file pone.0208537.s008.pdf]

**PKB/Akt: 58 kDa**

Control

HCD

Control+ART

HCD+ART

B-tubulin →

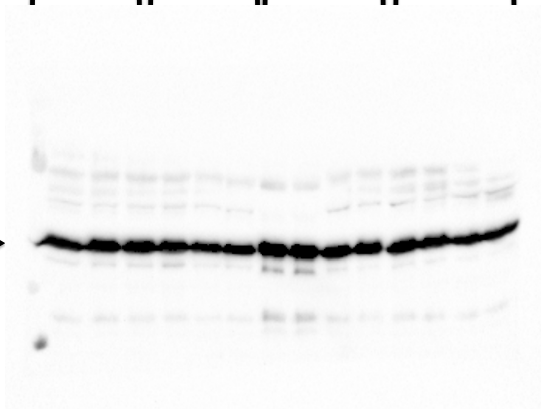

Supplement: S9 Fig — n = 3–4 /group. (Antibody: polyclonal; source: rabbit; dilution 1:1000) (PDF) [file pone.0208537.s009.pdf]

PKB/Akt: 58 kDa

Control      HCD      Control+ART      HCD+ART

Total PKB : C, HF (+/- ART), 1h repof.

Total PKB/Akt →

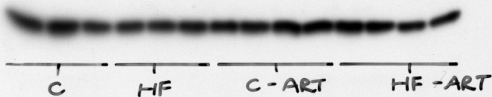

Supplement: S10 Fig — n = 3–4 /group. (Antibody: polyclonal; source: rabbit; dilution 1:1000) (PDF) [file pone.0208537.s010.pdf]

PKB/Akt: 58 kDa

Control

HCD

Control+ART

HCD+ART

Ponceau →

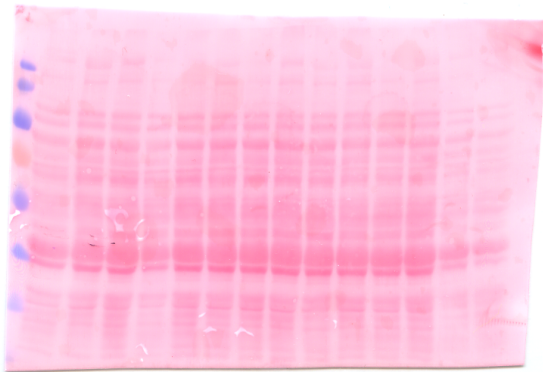

Supplement: S12 Fig — n = 3–4 /group. (PDF) [file pone.0208537.s012.pdf]

**AMPK: 62 kDa**

Control

HCD

Control+ART

HCD+ART

Total AMPK →

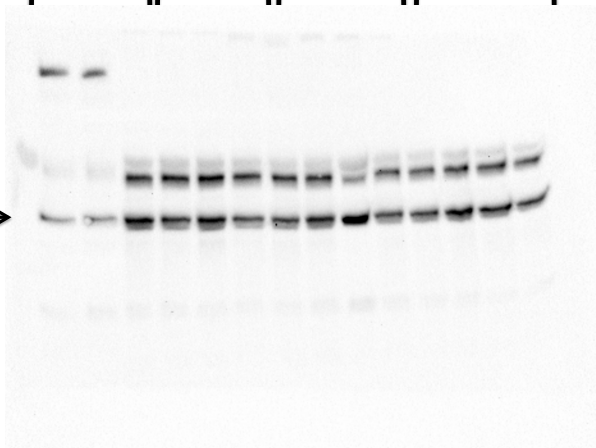

Supplement: S13 Fig — n = 3–4 /group. (Antibody: polyclonal; source: rabbit; dilution 1:1000) (PDF) [file pone.0208537.s013.pdf]

**AMPK: 62 kDa**

Control

HCD

Control+ART

HCD+ART

Phospho-  
AMPK →

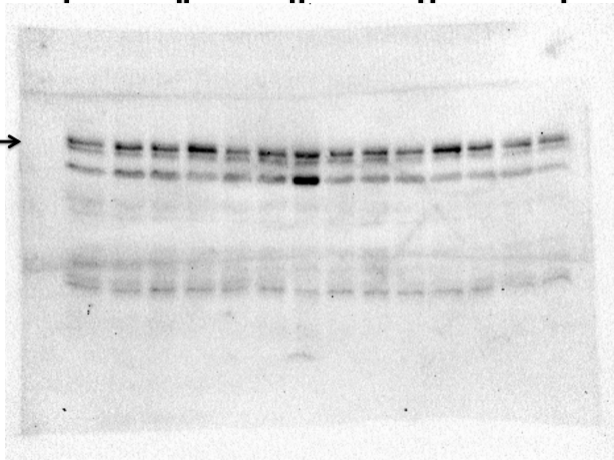

Supplement: S14 Fig — n = 3–4 /group. (Antibody: monoclonal; source: rabbit; dilution 1:1000) (PDF) [file pone.0208537.s014.pdf]

**AMPK: 62 kDa**

Control

HCD

Control+ART

HCD+ART

B-tubulin

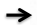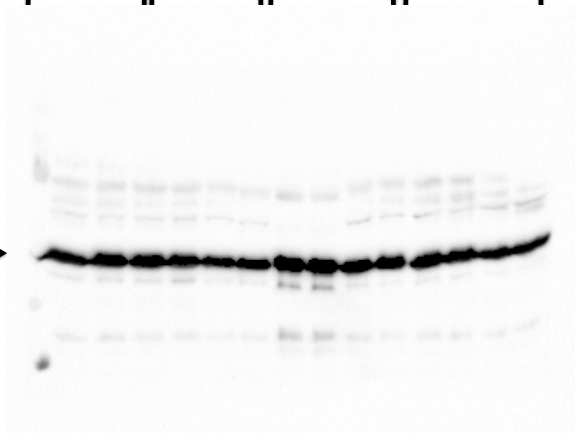

Supplement: S15 Fig — n = 3–4 /group. (Antibody: polyclonal; source: rabbit; dilution 1:1000) (PDF) [file pone.0208537.s015.pdf]

**AMPK:62 kDa**

Control

HCD

Control+ART

HCD+ART

Total AMPK

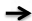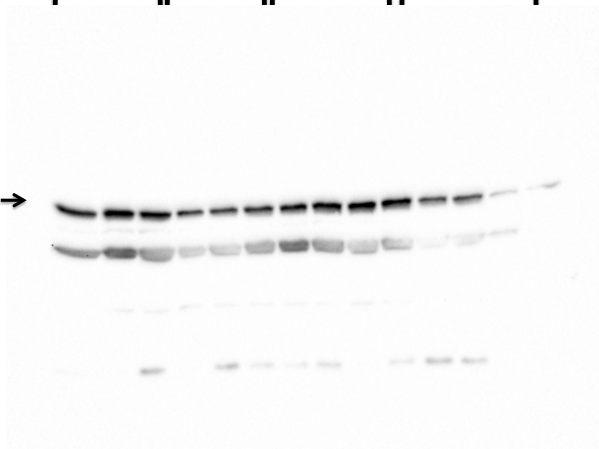

Supplement: S16 Fig — n = 3–4. (Antibody: polyclonal; source: rabbit; dilution 1:1000) (PDF) [file pone.0208537.s016.pdf]

**AMPK:62 kDa**

Control

HCD

Control+ART

HCD+ART

Phospho-  
AMPK

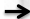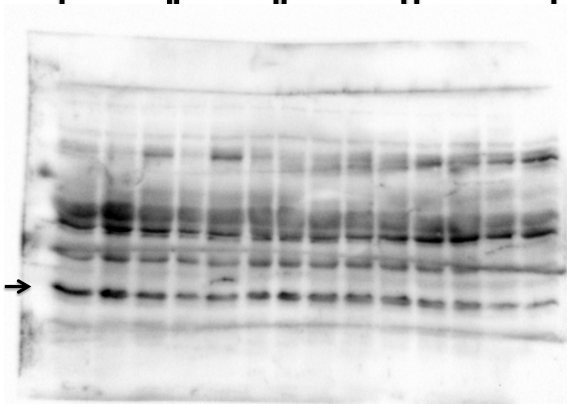

Supplement: S17 Fig — n = 3–4 /group. (Antibody: monoclonal; source: rabbit; dilution 1:1000) (PDF) [file pone.0208537.s017.pdf]

**AMPK:62 kDa**

Control      HCD      Control+ART      HCD+ART

Ponceau →

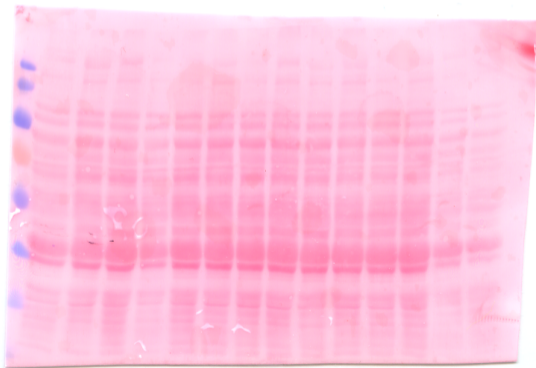

Supplement: S18 Fig — n = 3–4 /group. (PDF) [file pone.0208537.s018.pdf]

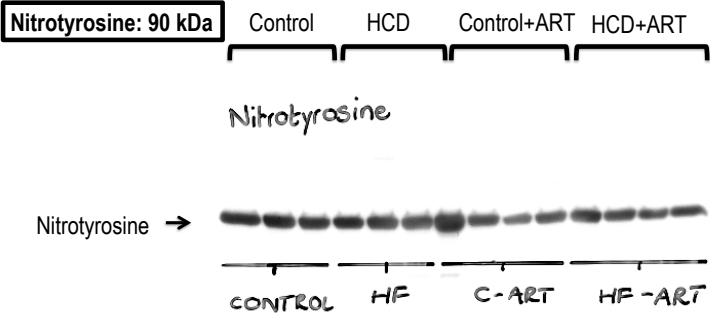

Supplement: S19 Fig — n = 3–4 /group. (Antibody: polyclonal; source: rabbit; dilution used 1:200) (PDF) [file pone.0208537.s019.pdf]

**Nitrotyrosine: 90 kDa**

Control

HCD

Control+ART

HCD+ART

B-tubulin →

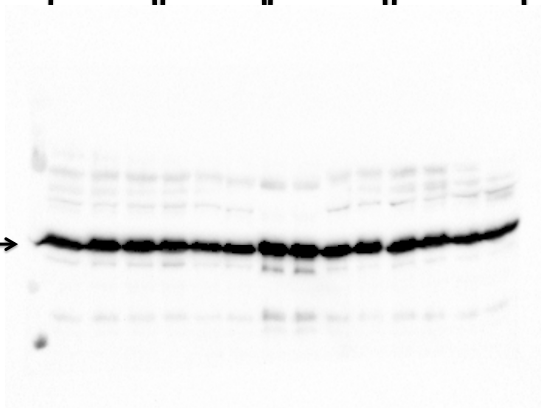

Supplement: S20 Fig — n = 3–4 /group. (Antibody: polyclonal; source: rabbit; dilution 1:1000) (PDF) [file pone.0208537.s020.pdf]

**Nitrotyrosine :62 kDa**

Control      HCD      Control+ART      HCD+ART

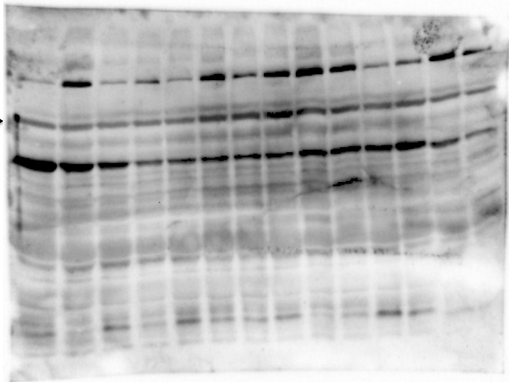

Nitrotyrosine →

Supplement: S21 Fig — n = 3–4 /group. (Antibody: polyclonal; source: rabbit; dilution 1:200) (PDF) [file pone.0208537.s021.pdf]

**Nitrotyrosine :62 kDa**

Control      HCD      Control+ART      HCD+ART

Ponceau →

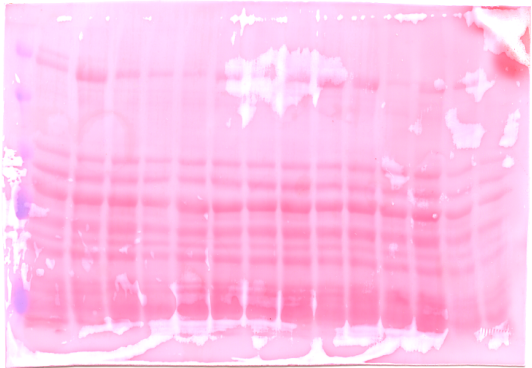

Supplement: S22 Fig — n = 3–4 /group. (PDF) [file pone.0208537.s022.pdf]

**P22 Phox: 22 kDa**

Control

HCD

Control+ART

HCD+ART

p22 Phox

P22 Phox

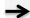

CONTROL

HF

C-ART

HF-ART

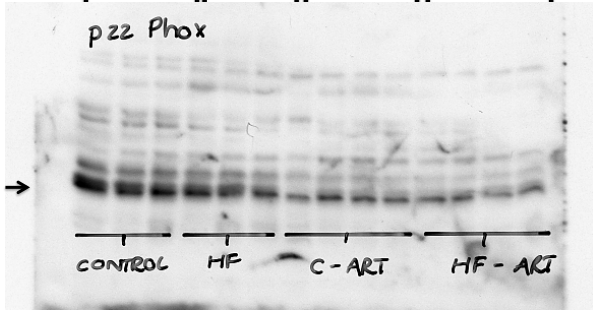

Supplement: S23 Fig — n = 3–4 /group. (Antibody: polyclonal; source: rabbit; dilution 1:200) (PDF) [file pone.0208537.s023.pdf]

**P22 Phox: 22 kDa**

Control

HCD

Control+ART

HCD+ART

B-tubulin

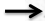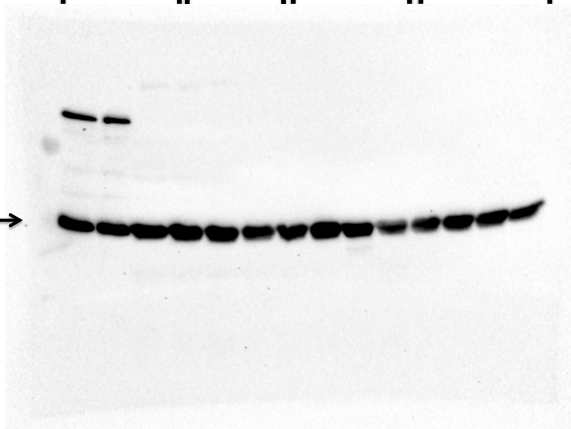

Supplement: S24 Fig — n = 3–4 /group. (Antibody: polyclonal; source: rabbit; dilution 1:1000) (PDF) [file pone.0208537.s024.pdf]

**P22 Phox: 22 kDa**

Control

HCD

Control+ART

HCD+ART

P22 Phox

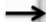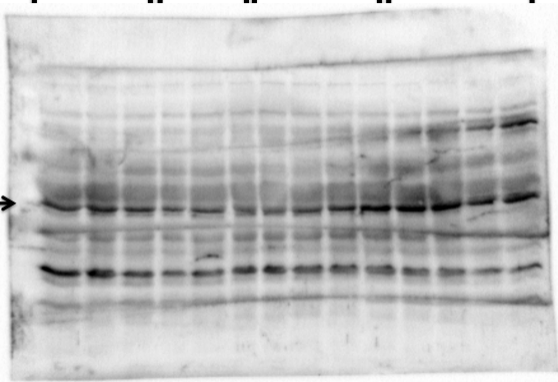

Supplement: S25 Fig — n = 3–4. (Antibody: polyclonal; source: rabbit; dilution 1:200) (PDF) [file pone.0208537.s025.pdf]

**P22 Phox: 22 kDa**

Control

HCD

Control+ART

HCD+ART

Ponceau →

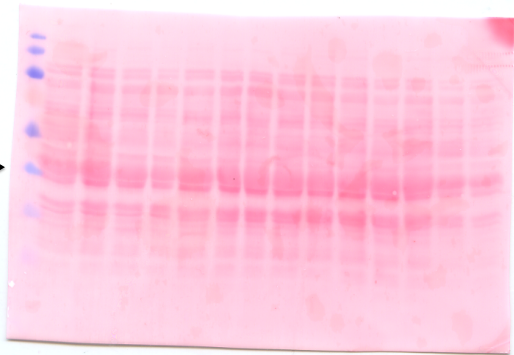

Supplement: S26 Fig — n = 3–4 /group. (PDF) [file pone.0208537.s026.pdf]
