# Supplementary material for: Treatment with a fixed dose combination antiretroviral therapy drug containing tenofovir, emtricitabine and efavirenz is associated with cardioprotection in high calorie diet-induced obese rats
Source: PLoS One. 2018 Dec 5;13(12):e0208537. doi: 10.1371/journal.pone.0208537 (PMC6281242; doi:10.1371/journal.pone.0208537)
Supplement: S4 Fig — n = 3–4 /group. (Antibody: polyclonal; source: rabbit; dilution 1:1000) (PDF) [file pone.0208537.s004.pdf]

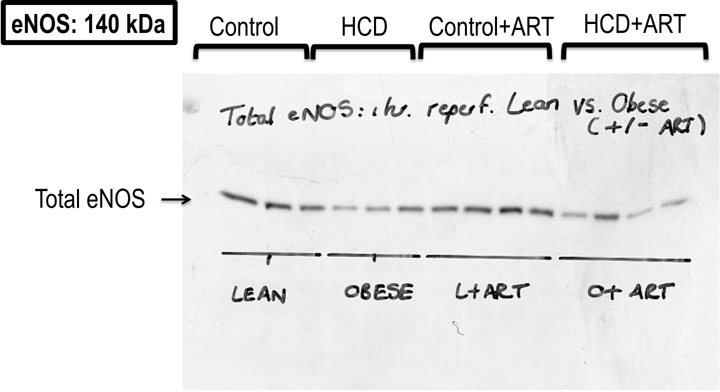
eNOS: 140 kDa
Control
HCD
Control+ART
HCD+ART
Total eNOS
Total eNOS: 4hr. reperf. Lean vs. Obese
(+/- ART)
LEAN
OBESE
L+ART
O+ ART
