# Supplementary material for: Treatment with a fixed dose combination antiretroviral therapy drug containing tenofovir, emtricitabine and efavirenz is associated with cardioprotection in high calorie diet-induced obese rats
Source: PLoS One. 2018 Dec 5;13(12):e0208537. doi: 10.1371/journal.pone.0208537 (PMC6281242; doi:10.1371/journal.pone.0208537)
Supplement: S11 Fig — n = 3–4 /group. (Antibody: monoclonal; source: rabbit; dilution 1:1000) (PDF) [file pone.0208537.s011.pdf]

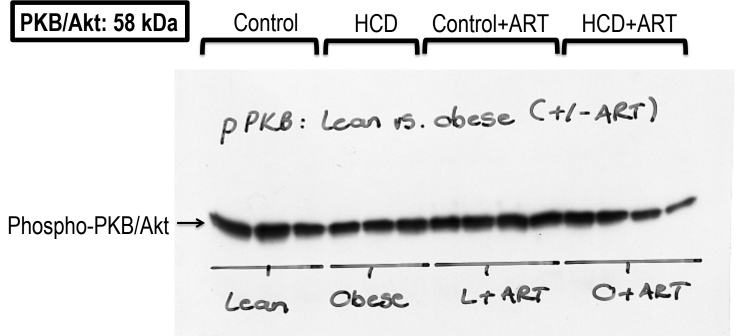
PKB/Akt: 58 kDa
Control
HCD
Control+ART
HCD+ART
pPKB: Lean vs. obese (+/-ART)
Phospho-PKB/Akt
Lean
Obese
L+ART
O+ART
